# Revealing the role of liquid preordering in crystallisation of supercooled liquids

**DOI:** 10.1038/s41467-022-32241-z

**Published:** 2022-08-04

**Authors:** Yuan-Chao Hu, Hajime Tanaka

**Affiliations:** 1 Department of Fundamental Engineering, Institute of Industrial Science, University of Tokyo, 4-6-1 Komaba, Meguro-ku, Tokyo 153-8505 Japan; 2 Research Center for Advanced Science and Technology, University of Tokyo, 4-6-1 Komaba, Meguro-ku, Tokyo 153-8904 Japan

**Keywords:** Phase transitions and critical phenomena, Phase transitions and critical phenomena, Structure of solids and liquids, Structure of solids and liquids, Chemical physics

## Abstract

The recent discovery of non-classical crystal nucleation pathways has revealed the role of fluctuations in the liquid structural order, not considered in classical nucleation theory. On the other hand, classical crystal growth theory states that crystal growth is independent of interfacial energy, but this is questionable. Here we elucidate the role of liquid structural ordering in crystal nucleation and growth using computer simulations of supercooled liquids. We find that suppressing the crystal-like structural order in the supercooled liquid through a new order-killing strategy can reduce the crystallisation rate by several orders of magnitude. This indicates that crystal-like liquid preordering and the associated interfacial energy reduction play an essential role in nucleation and growth processes, forcing critical modifications of the classical crystal growth theory. Furthermore, we evaluate the importance of this additional factor for different types of liquids. These findings shed new light on the fundamental understanding of crystal growth kinetics.

## Abstract (web summary)

In classical nucleation theory, structural order in the liquid phase is not considered. But simulations of supercooled liquids now show that crystal-like liquid preordering play an essential role in nucleation and growth processes - calling for extensions of the classical theory.

## Introduction

Crystallisation is one of the most ubiquitous yet mysterious phase transitions in nature. Understanding its physical mechanism is of practical interest in many fields, such as atmospheric science, nanoscale electronics, protein engineering, drug production, and physical metallurgy. Crystallisation usually proceeds by nucleation and subsequent growth, as described most famously by the classical nucleation theory (CNT)^1^. According to CNT, the equilibrium crystalline phase is nucleated randomly in a homogeneous disordered liquid by density fluctuations. Once the size of nuclei exceeds the critical size, they grow. The key thermodynamic factors for crystal nucleation are the free energy gain upon crystallisation, i.e., the chemical potential difference between the liquid and crystal phase Δ*μ*, and the free energy cost proportional to the interfacial tension *γ* associated with the formation of a new interface between the two phases. The balance of these two factors determines the critical nucleation free-energy barrier Δ*G* and the critical nucleus size *R*_c_.

Although CNT serves as an essential framework to understand crystallisation, the discovery of non-classical pathways for crystallisation challenges its general validity^2–11^. This is mainly because of the assumptions involved in the simple CNT. For example, it was argued that the crystal phase nucleated from the liquid is not necessarily the thermodynamically most stable one but can be the one having minimal free energy difference with the liquid phase (known as the Ostwald step rule)^12^ or the one with the lowest free energy barrier of formation^13^. It was also claimed based on the Landau theory that the body-centred cubic (bcc) phase should be nucleated preferentially and then transformed to the stable phase in simple atomic liquids^14^. Such two-(or multiple-)step crystallisation has been widely reported from numerical simulations and experiments, but still with controversy^2,3,6,15^. We may regard these scenarios as approaches from the crystal side.

On the other hand, recent studies of structural properties of glass-forming liquids^16^ have revealed that the supercooled liquid state is no longer homogeneous as assumed by CNT, and crystal-like angular order spontaneously develops in a supercooled state for systems suffering from only weak frustration against crystallisation. Such structural fluctuations have been found to play a dominant role in crystal nucleation, for example, by unsupervised learning methods^17^. That is, crystallisation begins with the enhancement of crystal-like bond orientational order, and then translational (density) ordering follows^4,7,10,16,18,19^. Thus, crystal nuclei are not born randomly but induced in regions of high crystal-like bond-orientational order in a supercooled liquid. In other words, the crystalline phase prefers to nucleate from preordered regions with local orientational symmetry consistent with the crystal. We may say that there is essentially no homogeneous nucleation in a strict sense. Nucleation always occurs through the “continuous” ordering, starting from orientational and then positional one^16,19^.

So far, this scenario has been mainly confirmed for relatively simple liquids with pair-additive isotropic potentials, such as hard spheres. How generally it is valid for more complex liquids such as metallic alloys is one issue. However, a much more crucial question is the role of such liquid structural preordering in crystal growth. Recently, fast crystal growth far beyond the prediction of CNT has attracted considerable attention^20–24^. According to classical crystallisation theory, the crystal growth rate is determined only by the diffusion constant *D* and the driving force of crystallisation Δ*μ*, independently of *γ*. Considering preordering of supercooled liquids, it is not clear at all whether this is true.

In this work, to address these fundamental issues, we carry out extensive molecular dynamics (MD) simulations with NiAl (Ni_50_Al_50_) as a model metallic alloy. The benefits of NiAl are two-fold: (1) its equilibrium crystal structure is B2, which is a bcc (body-centred cubic)-like structure consisting of two simple cubic interpenetrating sublattices; (2) spontaneous crystallisation is observable in the computational timescale. We find that the supercooled metallic liquid exhibits a non-classical crystallisation pathway, generalising the findings from hard-sphere-like systems with pair-additive potentials. More importantly, we show that both crystal nucleation and growth are mediated by local bond orientational order fluctuation rather than density fluctuation. Notably, by developing a novel order-killing strategy (OKS), we can control the degree of structural ordering in a supercooled liquid and consequently manipulate the crystallisation kinetics. Surprisingly, this method allows us to tune not only crystal nucleation but also growth rates to a large extent. We further show that the influence of structural ordering on crystallisation is through tailoring the liquid-crystal interfacial energy. This finding unambiguously demonstrates the crucial role of liquid preordering in crystallising metallic systems (including both nucleation and growth). The finding that liquid preordering affects crystal growth indicates the importance of the interface tension in crystal growth, unlike the prediction of the classical theory. Thus, an essential modification to the classical theory of crystal growth is necessary. We phenomenologically add the interfacial energy-related factor for estimating the impact of structural ordering on the growth rate. Then, we evaluate this factor for eight different systems with different bondings and crystal structures and discuss the implication of our results from the structural order type developed in these liquids.

## Results

### Structural orderings during crystallisation

We start by studying the spontaneous crystallisation process in NiAl by annealing the supercooled liquid at the nose temperature *T*_nose_ of its Time-Temperature-Transformation (TTT) curve (see Methods, section ‘MD simulations of NiAl crystallisation’). Since the nucleation event itself is stochastic (Supplementary Fig. 1a), we randomly follow one trajectory but find similar results from independent simulations (Supplementary Fig. 2). The local structural features are characterised by bond orientational order parameters (see Methods, section ‘Structural characterisation’).

**Fig. 1:**
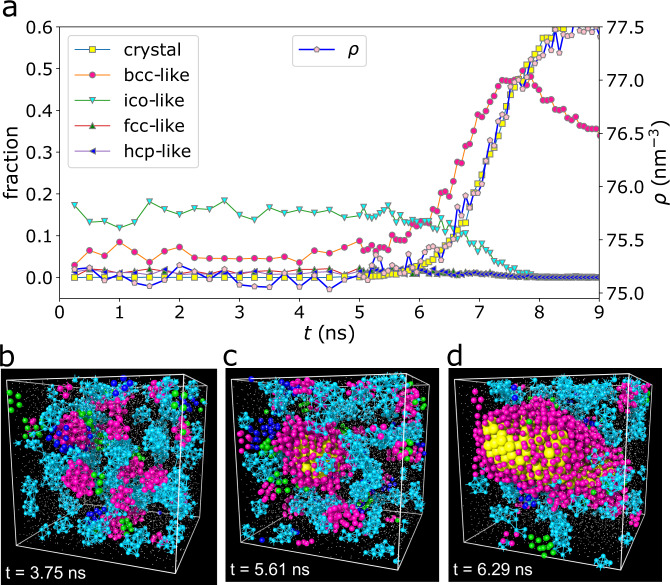
The nonclassical crystallisation process of NiAl at *T*_nose_. **a** Time dependence of the fraction of crystallised atoms (crystal) and various structural orderings. Bcc-like local orientational ordering is the preordering linked to the crystal in this system because the symmetry is compatible with the equilibrium crystal. Fcc-like and hcp-like preorders are almost negligible. There are always more atoms with ico-like order than those with crystal-like orders before crystallisation happens, i.e., in the incubation period. The temporal evolution of number density *ρ* is also included, which almost perfectly coincides with that of the crystallised atoms. **b**–**d** Atomic configurations at different time *t* (as indicated in each panel) in **a**. The atomic sizes are adjusted for visualisation. The atoms with orders are coloured corresponding to the legend in **a**. Both the centre and its nearest neighbours are shown for each unit of structural order. The other small dots are disordered liquid atoms. Preorders are transient and fluctuating in space, whereas crystal nuclei are born and grow from the bcc-like preordered regions. The crystal in **c** corresponds to the critical nucleus in this condition.

Figure 1a shows the time dependence of the fraction of crystallised atoms, together with that of preordered atoms with bcc-like symmetry. The time evolutions of other structural orders, with fcc-like, hcp-like, and ico-like symmetries, are also shown for comparison (fcc: face-centred cubic, hcp: hexagonal close-packed, ico: icosahedral). Here, bcc-like, fcc-like, and hcp-like orders are categorised as crystal-like since their symmetries are crystalline. We can find that the bcc-like one is dominant among these crystal-like orderings, and the others are almost negligible. It was proposed based on the Landau-type theory^14^ that the metastable precritical nuclei with bcc order are formed first, and then their cores transform to the stable crystalline (e.g., fcc) phase in the growth process. Such behaviour was indeed observed for a Lennard-Jones liquid^15^. This kinetic pathway is known as a non-classical (two-step) crystallisation pathway. In our case, bcc-like preorders formed in the supercooled liquid share the same symmetry with the stable B2 crystal phase, whereas the other crystal-like preorders (fcc-like and hcp-like) are negligible. Whether the bcc preordering is due to the Alexander-McTague scenario^14^ or preferred by the interaction potential is an interesting question. We think that the latter may be the case. Anyway, our observation excludes the formation of a metastable crystalline phase during crystallisation.

In Fig. 1, we can also see a plenty of ico-like clusters. This ordering may be favoured by the entropic effect since the icosahedral configuration provides high local packing capability, i.e., high vibrational entropy^25^. However, there is no quasicrystal formation mediated by ico-like preorders in our system, suggesting the strong internal geometrical frustration. Furthermore, we can see the strong competition between crystal-like and ico-like orderings from their mutually exclusive spatial distributions. Thus, ico-like orders only tend to prevent crystallisation without forming quasicrystals.

We can see crystallisation starting from approximately 5.0 ns. There are no crystallised atoms in the incubation period (*t* ≤ 5.0 ns), but always about 5% of the total atoms fluctuate as bcc-like preorders in the supercooled liquid. This conveys crucial messages. Firstly, the supercooled metallic liquid is not homogeneous as assumed by CNT but with local bond orientational order fluctuations heterogeneously in space. Secondly, these preorders are transient and have a finite lifetime. Thus, they are intrinsic to the supercooled liquid state. These features are similar to hard-sphere-like systems^4,18^ but still differ in that we find only bcc-like preordering in the metallic system^7^. This result can be understood as a consequence of the fact that preorders tend to share the symmetry of equilibrium crystals.

The atomic-scale features during crystallisation, as illustrated in Fig. 1b–d, provide further intriguing information. There are tiny preordered clusters fluctuating in space in the supercooled liquid state before crystal formation. The crystal nucleus is preferentially born within these preordered regions, initiated by local bond orientational order fluctuation but neither randomly as assumed by CNT nor by density fluctuation as assumed by the density functional theory. This is also revealed in Fig. 1a, where we can directly compare the number density change and bcc-like preorder. We can see that there is already a prominent amount of bcc-like preorder in the liquid state before crystal nucleation, which starts to increase before the onset of the density increase. We can see similar results in two other independent simulations (see Supplementary Fig. 2). We also stress that the local density field cannot detect these preordered regions (see Supplementary Fig. 3). So, we may say that the homogeneous nucleation of crystals occurs “heterogeneously in space”. More precisely, the crystal nucleation is mediated by crystal-like preorders spontaneously formed in a supercooled liquid state. Since the nucleated crystal phase is bcc-like, the same as the thermodynamically most stable one, it might look consistent with the classical scenario. However, the observation described above shows that crystallisation still proceeds in a two-step manner, more precisely, through sequential angular-then-positional ordering^4,16,18,19^. This fact indicates the generality of this physical picture to crystal nucleation in various materials, although each system has a specific preorder favoured by the interparticle interaction and entropy.

More importantly, Fig. 1 shows that the crystal nucleus grows while surrounded by preordered structures due to the interface wetting effect. This indicates a possibility that liquid preordering mediates not only crystal nucleation but also crystal growth. Therefore, the properties of the liquid-crystal interface can also be crucial in determining the crystal growth rate. However, such a possibility has been overlooked so far, e.g., in the Wilson-Frenkel (WF) theory^1,26,27^. Thus, we also focus later on how the preordering affects the crystal growth kinetics.

### Tuning crystal nucleation time

For unveiling the role of such liquid preordering in governing the crystallisation kinetics and elucidating the underlying physical mechanism, we develop the OKS method (see Methods, section ‘Order killing strategy’) to intentionally control the degree of preordering in the supercooled liquid state. In brief, we check the local bond orientational orders of the liquid at a time window of Δ*t*_MD_ and perform MD simulations iteratively with and without a harmonic bias potential. The bias aims to revert the targeted preorders to a disordered state, i.e., killing specific preorders. The shorter Δ*t*_MD_ more strongly suppresses the targeted preorders in a supercooled liquid. In this way, we can suppress local bond orientational order fluctuations in a controllable manner.

**Fig. 2:**
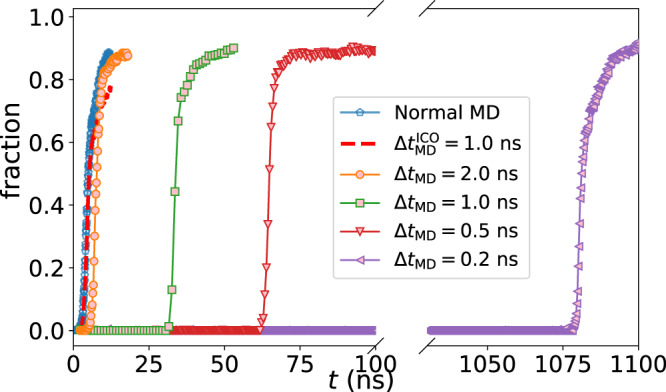
Crystal nucleation at *T*_nose_ tuned by OKS. The time dependence of the fraction of the crystallised atoms is shown at different conditions. Crystallisation originally starts from 2.0 ns during isothermal annealing (Normal MD). By killing the preordering periodically every Δ*t*_MD_ using the bias potential, the nucleation time has been remarkably changed. The shorter Δ*t*_MD_ is, the longer time is needed for nucleation to start. In contrast, the suppression of local icosahedral ordering ($\Delta {t}_{{{{{{{{\rm{MD}}}}}}}}}^{{{{{{{{\rm{ICO}}}}}}}}}=1.0$ ns, dashed line) shows little influence on the incubation time of crystal nucleation. All the biased simulations start from the same condition from Normal MD.

We first show how the nucleation time at *T*_nose_ is controlled by the structural orderings tuned by Δ*t*_MD_ in Fig. 2. Notably, crystal-like preordering plays a decisive role in initiating crystal nucleation. In standard isothermal annealing of the chosen crystallisation trajectory, nucleation begins from about 2.0 ns. However, when the fluctuation of the bcc-like ordering is suppressed, nucleation can be delayed considerably. The liquid with a shorter Δ*t*_MD_, that is, fewer bcc-like preorders, requires a longer time to nucleate the crystal phase. Remarkably, the time required for nucleation increases to 1078 ns when Δ*t*_MD_ is decreased to 0.2 ns. This incubation time is more than 500 times slower than the one in the normal condition. Further decrease in Δ*t*_MD_ would lead to even a longer incubation time.

Now, we consider the roles of ico ordering, which is another important type of ordering in our NiAl system. Topological orderings like local ico order in three dimensions are thought to be crucial in preventing crystallisation and glass formation from metallic liquids^28–33^, which can be dated back to Frank’s seminal work^34^. In addition to the number density, the spatial extendability of ico order has been suggested to be critical^35,36^. The formation of medium-range ico order, i.e., connected icosahedra, tends to suppress the development of crystal-like preorders more significantly than isolated icosahedra. The presence of spatially more extended ico ordering indicates its stronger ico ordering tendency, which overcomes internal structural frustration. Noting that crystallisation is controlled by thermodynamic competition between crystal-like and ico-like orderings, this means that crystallisation suffers from stronger frustration. Thus, the crystallisation kinetics should depend on the degree of such ico-like preorder suppression. It was shown by Desgranges and Delhommelle^36^ that this spatial icosahedral extendability may induce a non-monotonic temperature dependence of the ‘crystal’ nucleation barrier in supercooled metallic liquids. We also mention another possible influence of ico ordering on the fate of a liquid. When there is very strong tendency of ico ordering, their spatially extended connection may lead to the formation of metastable quasi-crystals^28,37^. Under such a circumstance, the ‘crystal’ nucleation is disfavored (the crystal nucleation barrier increases); instead ‘quasicrystal’ nucleation is favoured (the quasicrystal nucleation barrier decreases). Therefore, the spatial extent of ico-like order generally has significant effects on crystallisation. A critical factor is whether the symmetry is compatible with the (meta)stable phase to be formed or not.

In NiAl liquids studied here, when we eliminate the ico-like ordering instead of bcc-like ordering (see Methods, section ‘Order killing strategy’), the nucleation time is almost unchanged and comparable to the normal one (Fig. 2). Thus, we conclude that the suppression of crystal-like order by ico-like order is not significant; in other words, the crystal-like preorder primarily controls crystal nucleation (see Fig. 1c). This minor influence of periodically killing only ico-like orders on crystallisation is because crystals are nucleated exclusively in regions of high crystal-like preorders. This explains the negligible influence of the ico-like order killing on the crystal nucleation time. However, if there is much stronger tendency of ico-like ordering, it may affect the crystallisation. How significantly ico-like orderings affect the crystallisation kinetics should depend on how strongly they suppress the crystal-like orderings. This is an interesting topic for future study.

This result does not rule out the importance of icosahedra but rather helps unveil the mechanism of how icosahedra affect the crystallisation kinetics microscopically, as discussed above. Due to the incompatibility of local symmetry between icosahedra and crystal-like preorders, the appearance of icosahedra automatically suppresses and prevents crystal-like preordering. These two types of orders are mutually exclusive in a supercooled liquid. Therefore, the way by which icosahedra influences crystallisation is through suppressing preordering and changing the liquid-crystal interfacial energy^28,38^. We also note that in our simulations, both short-range and medium-range orders are considered. Because we focus on detecting the centres of crystal-like or ico-like preorders, we destroy these orders without differentiating whether they are isolated or connected. Separating the effects of short-range and medium-range orders is another interesting topic for future study.

### Tuning crystal growth rate

**Fig. 3:**
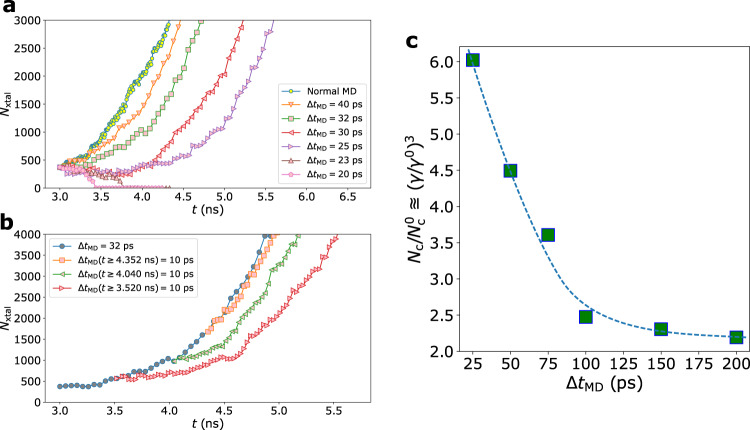
Crystal growth behaviour and the estimation of the critical nucleus size at *T*_nose_ tuned by OKS. **a**, The time dependence of the number of crystallised atoms *N*_xtal_ for different conditions by killing preorders. The crystal grows quickly in standard MD simulations of isothermal annealing (normal MD). Decreasing Δ*t*_MD_ reduces the crystal growth speed considerably without melting, thanks to the large size of the crystal seed. However, the seed can even be dissolved when the crystal-like preordering is strongly suppressed by very short Δ*t*_MD_. **b**, The time dependence of *N*_xtal_ in the following two-step killing procedures. We take crystallisation states from the case of Δ*t*_MD_ = 32 ps shown in **a**. Then, we reduce Δ*t*_MD_ to 10 ps at different growth stages, resulting in the more frequent removal of crystal-like preorders in this process. Since the larger crystal nucleus accompanies the larger amount of preorders, a shorter Δ*t*_MD_ is required to suppress the growth efficiently. The crystal growth rate can be effectively tuned by the degree of preordering through Δ*t*_MD_. **c**, The dependence of the normalized critical nucleus size ${N}_{{{{{{{{\rm{c}}}}}}}}}/{N}_{{{{{{{{\rm{c}}}}}}}}}^{0}$ and the normalized interfacial energy *γ*/*γ*^0^ on the degree of preordering (i.e., local bond order fluctuation) that is controlled by Δ*t*_MD_. Here ${N}_{{{{{{{{\rm{c}}}}}}}}}^{0}$ and *γ*^0^ are the corresponding values in the normal liquid state (without OKS). With shorter Δ*t*_MD_, there is less preordering, leading to the increase of *γ* effectively. The dashed curve is an eye guide.

Next, we investigate the role of preordering in crystal growth at *T*_nose_ by OKS (see Methods, section ‘Order killing strategy’). To study the rate of crystal growth, we first identify the critical nucleus size ${N}_{{{{{{{{\rm{c}}}}}}}}}^{0}$ in the normal state as 51 ± 15 by using the seeding method^38^. In the course of spontaneous crystallisation, configurations with various sizes of crystal seeds can be obtained. For example, we choose a seed of 375 atoms embedded in the supercooled liquid as the starting state. As shown in Fig. 3a, the number of the crystallised atoms *N*_xtal_ increases abruptly during normal isothermal annealing. However, when the preordering is suppressed via OKS, the crystal growth slows down remarkably. A shorter Δ*t*_MD_, i.e., more frequent order killing, leads to slower crystal growth. The crystal phase can even be destroyed if the preordering is strongly suppressed (see, e.g., the case of Δ*t*_MD_ shorter than 25 ps in Fig. 3a).

In order to further illustrate the crucial role of preordering in crystal growth, we use a two-step method to tune the structural ordering. As shown in Fig. 3b, we choose initial states with different crystal sizes from the simulations of Δ*t*_MD_ = 32 ps in Fig. 3a, and then we decrease Δ*t*_MD_ to 10 ps to kill the preordering more effectively. Obviously, more severe suppression of bcc-like preordering leads to the slower growth of the crystal. For the same Δ*t*_MD_, the impact of order killing is weaker for larger crystals. In other words, in the late stage of crystal growth, we need to use shorter Δ*t*_MD_ to slow down the growth because the preordering is prevailing around the large crystal seed (see Fig. 1). This result indicates the curvature dependence of the interface preordering, i.e., the liquid-crystal interfacial energy. This demonstrates that the interface preordering is of paramount importance also during crystal growth. If there is no preordering wetting the liquid-crystal interface, the order-parameter profile across the interface between the crystal and the liquid becomes very sharp. Such a significant structural difference across the crystal growth front, i.e., the large interface tension, makes the crystal growth into the liquid more difficult (see the additional simulations on Si below). This is intuitively natural since a more significant structural change at the growth front is required for crystals to grow. CNT has overlooked this fact.

### Critical nucleus size under the control of structural ordering

Inspired by the time dependence of *N*_xtal_ on Δ*t*_MD_ during crystal growth, we measure the critical nucleus size *N*_c_ under the conditions when preordering is suppressed by OKS (see Methods, section ‘Order killing strategy’). Figure 3c illustrates the dependence of the *N*_c_ normalized by ${N}_{{{{{{{{\rm{c}}}}}}}}}^{0}$ on the degree of preordering (i.e., local bond orientational order fluctuation) controlled by Δ*t*_MD_. Intriguingly, with decreasing Δ*t*_MD_, i.e., fewer preordering, ${N}_{{{{{{{{\rm{c}}}}}}}}}/{N}_{{{{{{{{\rm{c}}}}}}}}}^{0}$ increases more steeply, indicating the ascending critical nucleation barrier. Note that the value ${N}_{{{{{{{{\rm{c}}}}}}}}}/{N}_{{{{{{{{\rm{c}}}}}}}}}^{0}$ should plateau to 1.0 at large enough Δ*t*_MD_ eventually. The required time to reach the plateau will depend on the crystallisation time of the supercooled liquid. When Δ*t*_MD_ reaches the time when the crystal nucleus size exceeds the critical one, which we call the crystal nucleation time here, the order-killing will not affect the crystal nucleation process anymore. We estimated the average crystal nucleation time for NiAl at *T*_nose_ as 7.38 ± 5.61 ns from 10 independent simulations (see Supplementary Fig. 4). This is much longer than the largest Δ*t*_MD_ value (0.2 ns) used in Fig. 3c, leading to the unsaturation of *N*/*N*_0_. Since the driving force for crystallisation Δ*μ* is roughly unaltered (see Methods, section ‘Crystallisation driving force’), the rise of *N*_c_ should mainly originate from the increasing interfacial energy *γ*. In fact, CNT predicts the critical nucleus size as *R* = 2*γ*/(*ρ*_s_∣Δ*μ*∣), where *ρ*_s_ is the number density of the solid^1^. By assuming a spherical shape of the nucleus as in CNT, even though it is not necessarily to be the case, the change of *γ* normalised by the interface tension of the normal liquid state *γ*^0^ scales as the cube root of ${N}_{{{{{{{{\rm{c}}}}}}}}}/{N}_{{{{{{{{\rm{c}}}}}}}}}^{0}$. As a consequence, *γ*/*γ*^0^ is estimated to increase from approximately 1.30 for Δ*t*_MD_ = 0.2 ns to around 1.82 when Δ*t*_MD_ decreases to 25 ps (Fig. 3c). Therefore, the way by which preordering tailors the crystallisation kinetics is by adjusting *γ*. Both crystal nucleation and growth proceed via the non-classical pathway mediated by the local bond order because the emergence of preordering at the liquid-crystal interface can effectively reduce *γ* by wetting the crystal and lower the crystallisation barrier.

The correlation between *γ* and Δ*t*_MD_ shows the temperature (*T*) dependent nature of the interfacial energy, different from CNT in which it is assumed as constant. This is because CNT assumes that a liquid has a completely disordered structure. *γ*(*T*) can either decrease or increase with supercooling until the glass transition intervenes^39^. This feature depends on the local structural ordering characteristics. In systems where crystal-like preordering are prevalent, lowering *T* will enhance these crystal-friendly structures^16^ and reduce *γ*(*T*). This picture is consistent with the diffuse interface theory and has been confirmed by the studies on liquid metals^40–42^. The role of icosahedra in quasicrystal formation is similar to that of crystal-like preordering in ordinary crystallisation^28,29,37,43^. Such examples have been experimentally observed for ZrCu-based and Al-based bulk metallic glasses^44,45^. On the contrary, if local icosahedral order is dominant, but not strong enough for quasicrystal formation, and suppresses crystal-like preordering, *γ*(*T*) for crystal formation would increase and helps glass formation.

### Phenomenological modifications to the classical theory

We have shown that local structural orderings are crucial in crystallisation, but which have been overlooked in the classical theories. In the following, we make some essential modifications of CNT to incorporate the effect of such orderings. The “homogeneous” nucleation frequency of crystals in supercooled liquids is rewritten phenomenologically as

1 $$I(T)={C}_{{{{{{{{\rm{I}}}}}}}}}D\exp \left(-\frac{\alpha \gamma {(T)}^{3}}{\Delta \mu {(T)}^{2}{k}_{{{{{{{{\rm{B}}}}}}}}}T}\right),$$

where *C*_I_ is a constant prefactor, *D* is the translational diffusion coefficient normal to the crystal surface, *k*_B_ is Boltzmann constant and *α* is a factor accounting for the shape of the crystal nucleus. Both *γ* and Δ*μ* should have non-trivial temperature dependences. More importantly, *γ* should be closely related to the specific structural orders formed in a supercooled liquid and is determined by competing ordering effects^38,46^.

Regarding crystal growth, only two factors have been mainly considered traditionally: 1) the effective diffusion coefficient, which represents the atomic mobility towards the interface; 2) the driving force Δ*μ*, which dictates the willingness of an atom to join the crystal. However, based on the above findings of the critical role of structural orderings in crystallisation, we phenomenologically introduce a new factor *P*(*γ*(*T*)) to account for the microscopic details of the interfacial structural ordering. This factor measures how easily an atom finds a proper lattice position on the crystal surface. The rate of crystal growth is accordingly modified as

2 $$U(T)={U}_{{{{{{{{\rm{WF}}}}}}}}}(T)\cdot P\left(\gamma (T)\right)=\frac{6\ell }{{\lambda }^{2}}D\left[1-\exp \left(-\frac{\Delta \mu (T)}{{k}_{{{{{{{{\rm{B}}}}}}}}}T}\right)\right]\cdot P\left(\gamma (T)\right),$$

where *ℓ* and *λ* are respectively the inter-planar spacing and the displacement length based on the classical Wilson-Frenkel theory^1,26,27^. *U*_WF_(*T*) represents the Wilson-Frenkel theory without considering any effect from *γ*. We note that *U*_WF_(*T*) does not simply represent the crystal growth rate in the absence of preordering. It only considers the diffusion rate and thermodynamic driving force for crystallisation, neglecting the microscopic details of system-specific liquid-crystal interface properties. *U*_WF_(*T*) can strongly overestimate the crystal growth rate if the topological and chemical frustration effects are serious in a system. For example, in binary and multicomponent systems with large structural and chemical (compositional) gradients near the crystal surface, even without preordering, the crystal growth could be somewhat slower than the WF prediction. This may explain the discrepancy between experiments and theories previously reported^2,4^.

To solve this issue, we add a new factor *P*(*γ*(*T*)) incorporating the effect of the liquid structural ordering of a specific system on the kinetic and/or thermodynamic factors. In some sense, *P*(*γ*(*T*)) measures the wettability of the interface preordering to the crystal, even though its absolute value does not directly give the degree of wettability. We stress that it contains the kinetic effects. A large *P*(*γ*(*T*)) above unity indicates that crystal-like preorders wet the crystal. Thus, the WF theory underestimates the crystal growth rate. This scenario might explain the ultrafast crystal growth in pure metals far exceeding the prediction of the classical theories^21^. However, the meaning of *P*(*γ*(*T*)) < 1.0 should depend on the actual situation. This may be related to the temperature dependence of *γ* in a system^39^. If *γ*(*T*) decreases with increasing degree of supercooling, more crystal-like preordering could form when the temperature is lowered. Thus, the crystal phase should be more wetted. So, even when *P*(*γ*(*T*)) < 1.0, there may be wetting of crystals by preorders. However, if there is no preordering during cooling, *P*(*γ*(*T*)) < 1.0 tells no wettability. This is because if the wetting effect exists, the crystal growth should be accelerated.

**Fig. 4:**
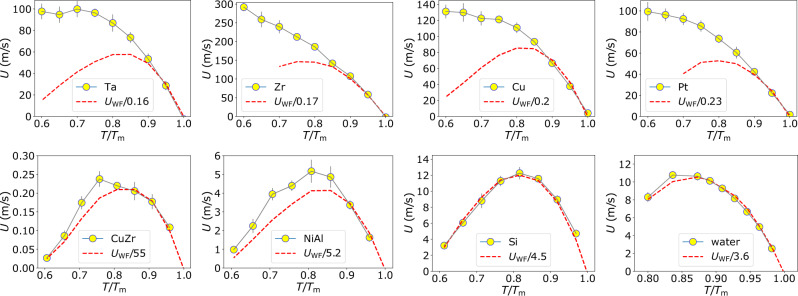
Comparison of the measured crystal growth rates with the predictions of the WF theory for various systems. The crystal growth rate *U* is measured from the planar liquid-crystal interface for each system. The WF prediction *U*_WF_(*T*) is calculated from equation (2) without considering *P*(*γ*(*T*)). A system-dependent constant is used to scale *U*_WF_(*T*) for better comparison. The WF values significantly overestimate and underestimate the crystal growth rates, depending on specific structural orderings. (The error bars represent SD).

**Fig. 5:**
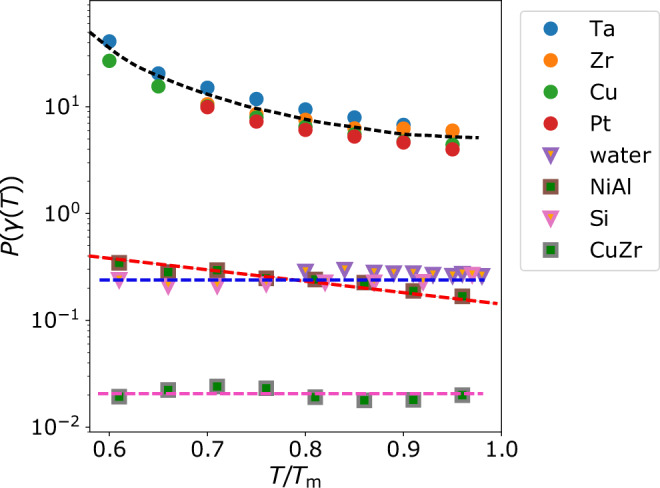
*P*(*γ*(*T*)) in supercooled states for different systems. They are measured by the scaling of the realistic crystal growth rate by the theoretical prediction, *U*(*T*)/*U*_WF_(*T*). CuZr has the smallest *P*(*γ*(*T*)) among these systems, which may originate from strong topological and compositional frustrations at the liquid-crystal interface. The dashed lines are guides to the eye, indicating the different trends of *P*(*γ*(*T*)) with supercooling.

To verify our idea, we directly calculate the variables in equation (2) to estimate the WF prediction and the actual crystal growth rate from MD simulations (see Methods, section ‘Crystal growth rate measurement’). The properties of the eight systems studied are listed in Table 1. The systems cover from single-component systems to binaries, metals to metalloids, isotropic interactions to anisotropic interactions, and three-body interactions to many-body interactions. They also have different equilibrium crystal structures and different types of locally favoured structures. Thus, the systems have enough variety for a general discussion valid to a broad range of materials. We show in Fig. 4 the temperature dependence of the measured crystal growth rate of each system (solid symbols), together with the values of the WF prediction (red dashed line). The WF theory fails seriously in describing the measured crystal growth rate. It underestimates *U* of monoatomic metals (Ta, Zr, Cu and Pt) and overestimates the ones of binaries and tetrahedral systems (CuZr, NiAl, Si, and water). The former case has also been verified in several fcc-forming metals^21^. In addition, besides water and Si, the temperature dependence of the WF prediction is distinctly different from the measured values. We show the temperature dependence of the correction factor *P*(*γ*(*T*)), which measures the discrepancy between the WF prediction and the measured *U* value, in Fig. 5. This result suggests that *γ* depends on the supercooling degree, as inferred from Fig. 3c. Increasing *P*(*γ*(*T*)) with lowering temperature implies a decreasing *γ* with supercooling. In the metallic systems, crystal-like bond orientational ordering formed in the supercooled state, as revealed in NiAl above, should help crystal growth. This effect is especially prominent in monoatomic liquid metals, which do not suffer from chemical (compositional) frustrations^38,47–50^. For Si and water, the local tetrahedral order will facilitate the growth of the diamond cubic crystal phase. These wetting effects originate from the compatible local symmetry between the preordering and the crystal phase.

The temperature dependence of *P*(*γ*(*T*)) in Fig. 5 also shows some interesting features. On the one hand, there is a broad range of *P*(*γ*(*T*)) for these systems, demonstrating different characteristics of liquid structural orderings when assisting crystal growth. On the other hand, the dependence of *P*(*γ*(*T*)) on the degree of supercooling is significantly different among the systems. *P*(*γ*(*T*)) increases when more supercooled for monoatomic metals. The binary NiAl behaves somewhat similar to metals but exhibits a weaker increase of *P*(*γ*(*T*)) with decreasing *T* than metals. However, for CuZr, Si, and water, *P*(*γ*(*T*)) keeps almost constant at various degrees of undercooling. We analyse the structural orderings in the equilibrated supercooled states and at the crystal-growth front for Zr, Ta, NiAl, CuZr, and Si to rationalise these observations. First of all, the degree of the spatial correlation of crystal-like preordering is important in crystal growth. A stronger correlation would help crystal growth more strongly. We compare the spatial correlation of the bond orientational order parameter (*Q*_6_ and *Q*_12_) in Supplementary Fig. 11 and find a substantial increase in the spatial correlation of the preordering upon cooling for metals. Such correlation is weaker for NiAl and even weaker and almost temperature independent for CuZr. In Si, the spatial correlation of the order parameter is nearly absent.

**Fig. 6:**
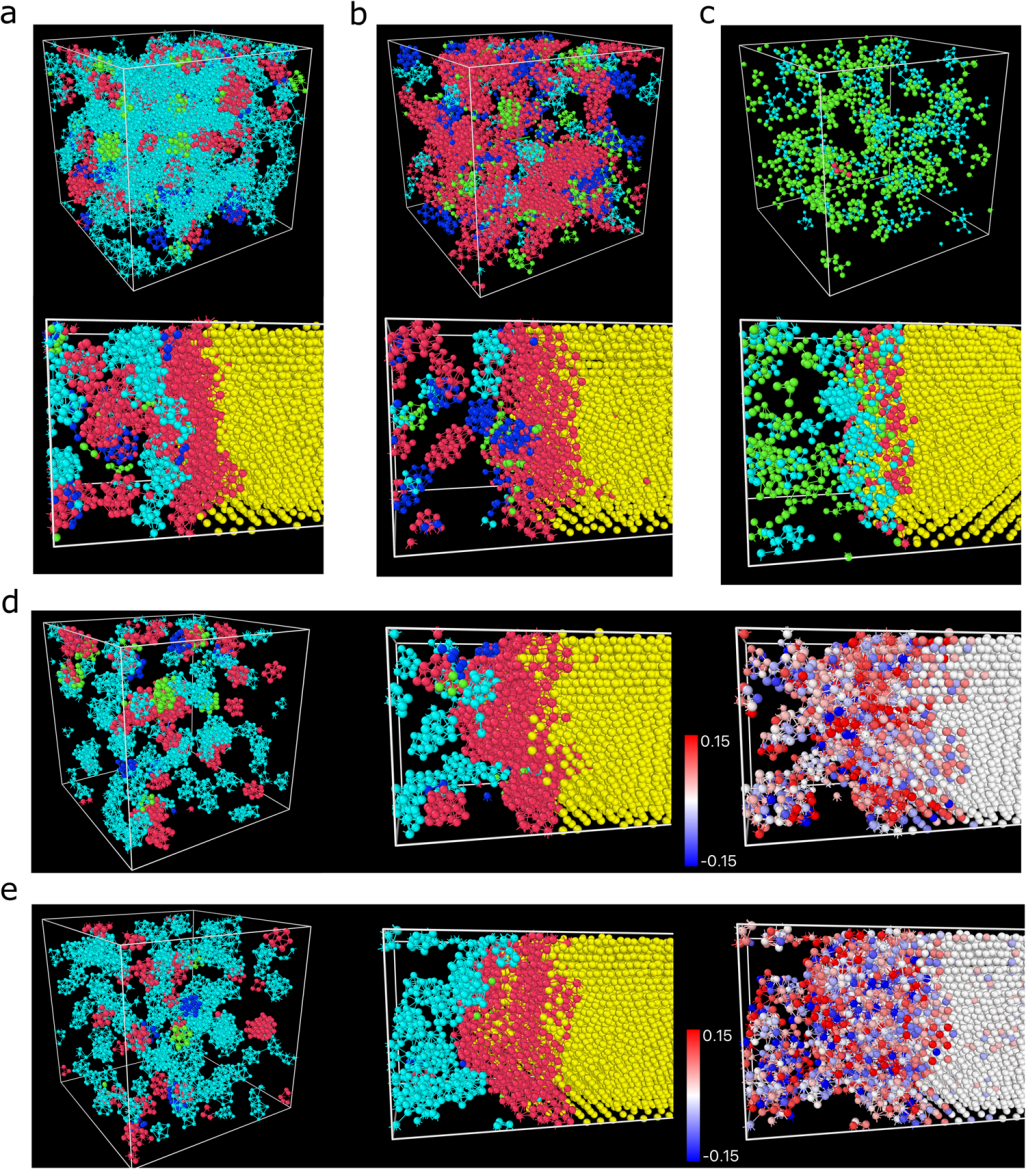
Structural orderings in supercooled states and crystal growth fronts of various systems at 0.7*T*_m_. The systems in **a**–**c** are Ta, Zr and Si, respectively. For each of them, the upper panel is the structural orderings in the equilibrated supercooled liquid, while the lower panel shows the structural orderings at the liquid-crystal interface during crystal growth. The systems in **d**, **e** are NiAl and CuZr, respectively. For each of them, the left panel depicts the structural orderings in the equilibrated liquid state, the middle panel shows the structural orderings at the liquid-crystal interface during crystal growth, and the right panel illustrates the distribution of the deviation of the local chemical composition from the B2 lattice. The atoms are coloured by (*c*_*α*_ − 6/14), where *c*_*α*_ is defined as the fraction of *α*-type atoms in the nearest neighbours of a central *α*-type particle (the white atoms are crystals). In the cases of crystal growth, only a part of the systems is shown for clarity. For the metallic systems, the pink, cyan, green, and blue colours represent bcc-like (preorder), ico-like, fcc-like, and hcp-like structural orderings, respectively. 14 or 12 nearest neighbours are shown for bcc-like or ico- or fcc- or hcp-like orders, respectively. In Si, the pink atoms have 10-11 connected neighbours, the cyan atoms have 6–9 connected neighbours, and the green atoms have 5 connected neighbours. Four neighbouring atoms are included for visualisation (i.e. forming a tetrahedral, for example). In all systems, the yellow atoms denote crystals. The sizes of particles have been adjusted for better visualisation. The normal liquid atoms are not shown.

Secondly, we show the spatial distribution of various structural orderings at 0.7*T*_m_, below which Zr is hard to equilibrate without crystallisation, in Fig. 6. As we can see, in the supercooled state, there are lots of ico-like orderings in Ta, while bcc-like preordering is dominant in Zr (Fig. 6a, b). Both show rich preordering at the liquid-crystal interface. However, there are much more ico-like orders adjacent to crystal-like preorders in Ta than Zr. The ico-like ordering should hinder the growth of crystal-like preorders into the liquid region. This feature explains the slower crystal growth rate (Fig. 4) and better glass-forming ability of Ta than Zr^51^. As shown in Fig. 6d, similar to Fig. 1b, some ico-like orders are coexisting with bcc-like preorders in the supercooled state. This situation is similar to CuZr, but which is more abundant of ico-like orders while the bcc-like preorders become rarer and more dispersed in space. This relationship between NiAl and CuZr is similarly found for the crystal-growth front structure (Fig. 6d, e, middle panel). Even though the bcc-like preorders wet the interfacial crystal in CuZr, the large amount of ico-like orders adjacent to the interface would impede crystal growth. Meanwhile, both NiAl and CuZr suffer from compositional frustration^38,47–50^ (see also Supplementary Fig. 12), which is even more severe in the latter (Fig. 6d, e, right panel). The bond strength between atoms should be very critical in determining the local composition adjustment rate^38^, which is directly linked to the crystal growth rate. Furthermore, the slower-growing speed, i.e., smaller *P*(*γ*(*T*)), of NiAl than the monoatomic metals (see Fig. 5) should originate from more ico-like orders and more substantial compositional frustration at the interface fronts in NiAl than these metals. This demonstrates the significant role of chemical-composition fluctuation in crystal growth, an intrinsic, unique feature of multicomponent (metallic) systems. This provides a physical explanation for the increase in the glass-forming ability of metallic alloys with the number of components. As for the tetrahedral system like Si (Fig. 6c), the preorders are much fewer and more independent from each other in space than monoatomic metals. Therefore, the liquid-crystal interface is much sharper. There is a steep gradient in the structural order parameter at the interface, without composition fluctuation, which makes *P*(*γ*(*T*)) small and independent of temperature change.

Thus, the difference of *P*(*γ*(*T*)) among these systems indicates that the crystal growth rate is determined by the degree of crystal-like preordering on the crystal surface and, additionally, the compositional fluctuation for the binary systems. All these microscopic features contribute to determining the liquid-crystal interfacial energy *γ*. How quantitatively each of these factors determines *γ* is an interesting topic for future study. These results strongly indicate the crucial role of interfacial preordering and interfacial energy in the crystal growth kinetics.

## Discussion

Our findings suggest that the interface tension *γ*(*T*) could be the most crucial factor in governing the crystallisation kinetics and glass-forming ability of various substances, including metallic glasses^20,38,46^. It means that the glass-forming ability is microscopically governed by the competing ordering effects^16^. Considering the case in NiAl, with Δ*t*_MD_ = 0.2 ns, the increase of *γ*(*T*_nose_) only by a factor of 1.30 leads to a delay of crystallisation by nearly three orders of magnitude. As is well known, the crystallisation time at *T*_nose_ is the shortest and directly determines the critical cooling rate. Therefore, suppressing the fluctuation of local bond orientational order friendly to the crystal improves the glass-forming ability significantly. Further enhancement of *γ*(*T*_nose_) can even turn a poor glass-former into an excellent one. Our findings also indicate the different temperature dependences of *γ* in different materials, which may provide another crucial piece for improving CNT and better understanding the glass-forming ability in metallic glasses.

In conclusion, we have studied the crystallisation mechanism in a supercooled metallic liquid and found that crystal forms and grows via non-classical pathways. Both crystal nucleation and growth are mediated by local crystal-like bond orientational order fluctuation, seriously questioning the assumptions of the classical theories. By developing the new order-killing technique, we successfully manipulate the crystallisation kinetics to a large extent, even at the same thermal condition. The degree of liquid preordering controls crystallisation by tailoring the liquid-crystal interface’s properties. This indicates the crucial role of interfacial energy in crystallisation and glass formation. We have shown that an essential modification to the classical theory is necessary, evaluated the correction factor, and discussed its meaning for various material types. Crystal-like preordering may influence the kinetic and/or thermodynamic factors. It is highly desirable to theoretically reveal the underlying physical mechanism of how preordering affects crystallisation kinetics. Our findings should deepen the fundamental understanding of the crystallisation mechanism in supercooled liquids and pave a new way to control crystallisation and glass formation.

## Methods

### MD simulations of NiAl crystallisation

We perform extensive MD simulations for NiAl by employing the many-body embedded atom method (EAM) potential^52^. All the simulations are run by using the open-source LAMMPS software^53^, in which periodic boundary conditions are kept in three directions and the time step for integral is 0.002 ps. Isobaric-isothermal ensemble (NPT) with zero external pressure is employed. We first create a bcc lattice with *N* = 8192 atoms and equilibrate it at a high temperature (2000 K). Then, we quench the system instantly to the desired temperature *T*_relax_ (mainly the nose temperature *T*_nose_ from the Temperature-Time-Transformation curve) for isothermal annealing and wait for crystallisation. The system size is designed so that there is only one critical nucleus during crystallisation. For NiAl, *T*_nose_ ≊ 921K(~0.6*T*_m_) (see Supplementary Fig. 1 and Ref. [54]). 8 independent simulations are generally carried out for all simulations, which give similar results. The simulation methods for other systems (see below) are essentially similar.

### Order killing strategy

To effectively control the structural (or, more precisely, local bond orientational order) fluctuations, we develop OKS by periodically performing biased MD simulations and normal MD simulations. The biased MD simulations are performed by using PLUMNED 2 patched to LAMMPS^55^. During the isothermal annealing, we check the local bond orders at a period of Δ*t*_MD_ (normal MD simulation timescale in each round). Suppose preorders are detected by bond orientational order parameter *Q*_6_ (see below). In that case, we perform MD simulations with a harmonic bias potential acting on these preorders to revert them to the disordered liquid state. This is realised by displacing the centre atoms. We emphasise that we do not bias crystallised atoms. If there are no preorders, we will run normal MD simulations. So Δ*t*_MD_ is key to control the degree of structural fluctuations. The shorter Δ*t*_MD_ is, the stronger the suppression of structural fluctuations will be.

To construct the bias potential, we first need to define a collective variable *q*_6v_ as the average *q*_6_(*i*) of the selected atoms. In the context of enhanced sampling of PLUMED 2, *q*_6_(*i*) is defined as

3 $${q}_{6}(i)=\sqrt{\mathop{\sum }\limits_{m=-6}^{6}{q}_{6m}{(i)}^{*}{q}_{6m}(i)},$$

where

4 $${q}_{6m}(i)=\frac{{\sum }_{j}\sigma ({r}_{ij}){Y}_{6m}({{{{{{{{\bf{r}}}}}}}}}_{ij})}{{\sum }_{j}\sigma ({r}_{ij})}.$$

*Y*_6*m*_ is the sixth order spherical harmonics and

5 $$\sigma ({r}_{ij})=\frac{1-{({r}_{ij}/{r}_{0})}^{50}}{1-{({r}_{ij}/{r}_{0})}^{100}}$$

is the switching function to determine whether atom *j* is the nearest neighbor of atom *i*. We choose *r*_0_ to be 3.5 Å for better efficiency. The bias potential takes the form of

6 $${V}_{{{{{{{{\rm{bias}}}}}}}}}=k{\left[({q}_{{{{{{{{\rm{6v}}}}}}}}}-a)/s\right]}^{2},$$

in which we set *k* = 10, *s* = 0.1 and *a* = 0. The restraining potential (UPPER_WALLS) starts acting on the preorderings when their *q*_6v_ is above 0 and tries to decrease it back to 0. The effective time of the bias potential is set as 10^4^ MD steps, which is large enough for energy dissipation in our case. In this way, we are able to suppress the structural fluctuations in a controllable manner. OKS is a little bit computational costly because we need to characterize the local structure frequently and calculate the collective variable every MD step during the biased simulations.

When we study crystal growth or measure the critical nucleus size under a bias, a crystal seed of a specific size is first generated in the supercooled liquid by normal MD simulations in spontaneous crystallisation. We then use OKS during the seeding simulations. The critical nucleus is identified when the probability of the crystal seed growing or dissolving becomes equal, i.e., when the crystal seed becomes stable. As described below, we identify the crystallised atoms through the bond orientational order methods for the crystal nucleation and growth processes.

The effective force to kill preorder from the implementation of *V*_bias_ may increase the temperature slightly in the liquid state. However, since the bias force is relatively weak, we confirm that such temperature effects on the crystal nucleation and growth are very weak (see Supplementary Figs. 5 and 6). Similar results are also observed for the fluctuations of pressure and number density (see Supplementary Fig. 7). Unlike the conventional biasing method, where one can clearly separate the free energy part and the kinetic factor part, our strategy involves the kinetic factor in the biasing. Nevertheless, we note that the influence of the bias potential on the atomic motion is negligible. As shown in Supplementary Fig. 8, the atom position is only perturbed inside its cage by the biasing. Thus, the periodic perturbation does not affect the rate factor, i.e., particle diffusion. Therefore, we can conclude that OKS only affects the thermodynamic factor through perturbating crystal-like orders in the liquid without affecting the kinetic factor.

### Structural characterisation

We use bond orientaional order parameters to define the local structures in supercooled metallic liquids^56^. The neighbours of each atom is defined by the radical Voronoi tessellation^57^. Firstly a complex vector for atom *i* is computed as

7 $${q}_{lm}(i)=\mathop{\sum }\limits_{j=1}^{{N}_{i}}{f}_{j}{Y}_{lm}(\theta ({{{{{{{{\bf{r}}}}}}}}}_{ij}),\phi ({{{{{{{{\bf{r}}}}}}}}}_{ij})),$$

where *N*_*i*_ is the number of the nearest neighbours of atom *i*, − *l* ≤ *m* ≤ *l*, and *Y*_*lm*_ is the spherical harmonics. *f*_*j*_ is the fraction of Voronoi polyhedral face area between *i* and *j* over the overall face area. The coarse-grained order parameter *Q*_*l*_ as

8 $${Q}_{l}(i)=\sqrt{\frac{4\pi }{2l+1}\mathop{\sum }\limits_{m=-l}^{l}{\left|{Q}_{lm}(i)\right|}^{2}},$$

in which *Q*_*lm*_(*i*) is the average of *q*_*lm*_(*i*) over the first coordination shell, including atom *i* itself. To detect the crystallised atoms in the metallic systems (Ta, Zr, Pt, Cu, NiAl and CuZr, see below), we define an order parameter

9 $${S}_{6}(i,\; j)=\frac{\mathop{\sum }\nolimits_{m=-6}^{6}{q}_{6m}(i){q}_{6m}^{*}(j)}{\sqrt{\mathop{\sum }\nolimits_{m=-6}^{6}{\left|{q}_{6m}(i)\right|}^{2}}\sqrt{\mathop{\sum }\nolimits_{m=-6}^{6}{\left|{q}_{6m}(j)\right|}^{2}}}.$$

In this calculation, we weighted the spherical harmonics of each atom from its nearest neighbours by their Voronoi face area, respectively^58^. The bond between *i* and *j* is treated as crystalline if *S*_6_(*i*, *j*) > 0.7, and if the number of crystalline bonds exceeds 10 we treat *i* as crystallised. As for NiAl, specifically, since the primary crystalline phase is B2 (bcc-like), we find there are no fcc-like or hcp-like structural orderings during crystallisation, which is different from the colloidal systems^7^. Therefore, the atoms with *Q*_6_ larger than 0.25 but with the number of crystalline bonds less than 10 are considered preorders (with bcc-like symmetry). We use the parameter *w*_6_ defined as

10 $${w}_{6}=\mathop{\sum}\limits_{{m}_{1}+{m}_{2}+{m}_{3}=0}\left(\begin{array}{ccc}6&6&6\\ {m}_{1}&{m}_{2}&{m}_{3}\end{array}\right){q}_{6{m}_{1}}{q}_{6{m}_{2}}{q}_{6{m}_{3}},$$

to identify the icosahedral center (*w*_6_ < − 0.023)^59^. The term in parentheses is the Wigner 3-*j* symbol. The different crystal-like structural orderings (bcc-like, fcc-like, hcp-like) are characterised by the order parameters

11 $${W}_{l}=\mathop{\sum}\limits_{{m}_{1}+{m}_{2}+{m}_{3}=0}\left(\begin{array}{ccc}l&l&l\\ {m}_{1}&{m}_{2}&{m}_{3}\end{array}\right){Q}_{l{m}_{1}}{Q}_{l{m}_{2}}{Q}_{l{m}_{3}},$$

in which *l* ∈ {4, 6}. A particle belongs to bcc-like crystal is indicated by *W*_6_ > 0, while for fcc-like crystalline atoms (*W*_6_ < 0, *W*_4_ < 0) and for hcp-like crystalline atoms (*W*_6_ < 0, *W*_4_ > 0).

Similarly, for tetrahedral systems (Si and water), we define *S*_12_(*i*, *j*) as the following to characterise the crystallised atoms^46^:

12 $${S}_{12}(i,\; j)=\frac{\mathop{\sum }\nolimits_{m=-12}^{12}{Q}_{12m}(i){Q}_{12m}^{*}(j)}{\sqrt{\mathop{\sum }\nolimits_{m=-12}^{12}{|{Q}_{12m}(i)|}^{2}}\sqrt{\mathop{\sum }\nolimits_{m=-12}^{12}{|{Q}_{12m}(j)|}^{2}}}.$$

We take the nearest 16 atoms as the particle neighbours in these calculations. An atom is treated as crystallised if it has more than 12 crystalline bonds (connected neighbours) as *S*_12_(*i*, *j*) > 0.75 for a pair.

To characterise the properties of the structural ordering in space, we calculate the spatial correlation function based on *Q*_*l*_ (*l* = 6, 12) as

13 $$\frac{{G}_{l}(r)}{g(r)}=\frac{4\pi }{2l+1}\frac{{\sum }_{ij}\mathop{\sum }\nolimits_{m=-l}^{l}{Q}_{lm}(i){Q}_{lm}^{*}(j)\delta ({r}_{ij}-r)}{{\sum }_{ij}\delta ({r}_{ij}-r)}.$$

Upon selecting preorders from the liquid state to kill, we choose the bcc-like preorders by *Q*_6_ > 0.25 in which the spherical harmonics are not weighted by the Voronoi face area. This simple method is effective because the fcc-like and hcp-like preorders are negligible (see Fig. 1). It is beneficial in saving computational time. On the other hand, the local icosahedral orderings can be automatically excluded (see Supplementary Fig. 9).

### MD simulations on additional systems

To study the crystal growth kinetics, we perform additional simulations on eight different systems: Ta, Zr, Cu, Pt, CuZr, NiAl, Si and water. The first six systems are modeled by the empirical many-body EAM potentials^51,52,60,61^, while the interatomic interactions in the latter two systems are described by the three-body Stillinger-Weber potentials^62,63^. All the simulations are run by using the open-source LAMMPS software^53^, in which periodic boundary conditions are kept in three directions, and the time step for integral is 5 fs for water and 0.002 ps for the others. The specific simulation strategies are designed for different measurements (see below). Generally, five independent simulations are performed for ensemble average.

### Crystallisation driving force

In our simulations, the thermodynamic driving force for crystallisation Δ*μ*(*T*) is estimated empirically via

14 $$\Delta \mu (T)=\Delta {H}_{{{{{{{{\rm{m}}}}}}}}}\left(\frac{{T}_{{{{{{{{\rm{m}}}}}}}}}-T}{{T}_{{{{{{{{\rm{m}}}}}}}}}}\right),$$

where Δ*H*_m_ is the enthalpy of fusion per atom at the melting temperature *T*_m_. To measure Δ*H*_m_(*T*_m_) we create equilibrium solid (crystal) and liquid at *T*_m_ respectively and calculate the enthalpy difference directly. We first create a cubic cell based on the specific material crystal structure (see Table 1). We generate 18 × 18 × 18 unit cells for bcc and B2 types, 14 × 14 × 14 unit cells for fcc type, and 11 × 11 × 11 unit cells for dc type. So roughly *N* ~ 11000 atoms are considered for each system. *NPT* (constant number *N*, constant pressure *P*, constant temperature *T*) emsemble is used for all the simulations. The simulation procedure consists of the following steps: (1) relax the initial configuration at a temperature much lower than *T*_m_; (2) heat the crystal to *T*_m_; (3) equilibrate the crystal at *T*_m_; (4) heat the crystal to a temperature much above *T*_m_; (5) equilibrate the melt at that high temperature; (6) cool the liquid to *T*_m_; (7) equilibrate the liquid at *T*_m_. The simulation time of step (5) is 4.0 ns, and it is 1.0 ns for the other steps. The enthalpy of the crystal and liquid is calculated from steps (3) and (7), respectively.

**Table 1:** Parameters of systems for the crystal growth rate measurements

	Ta	Zr	Cu	Pt	CuZr	NiAl	Si	water
*T*_m_ (K)	3285	2110	1355	1530	1340	1535	1450	275
Δ*H*_m_(*T*_m_) (eV/atom)	0.277	0.182	0.142	0.133	0.199	0.236	0.269	0.055
crystal structure	bcc	bcc	fcc	fcc	B2	B2	dc	dc

*dc* means diamond cubic

### Crystal growth rate measurement

We construct a planar liquid-crystal interface (see Supplementary Fig. 10 as an example) to calculate the crystal growth rate *U*(*T*) at various temperatures for the eight systems. A rectangular cell is designed for each system based on the material crystal structure (see Table 1). Specifically, we create 36 × 12 × 12 unit cells for bcc and B2 types, 30 × 10 × 10 unit cells for fcc type, and 24 × 8 × 8 unit cells for dc type. The liquid-crystal coexistent configuration is then generated by pinning part of the crystal while melting the others after equilibrating the initial crystal configuration. After relaxing the coexistent configuration at *T*_m_, the system will be annealed at the target temperature to monitor crystal growth. In the annealing process, *NPT* ensemble is used, but only the pressure along the elongated direction is controlled at 0, so only the box length along that direction can be adjusted for crystal growth and melting. The crystallisation process is analysed by using *S*_6_(*i*, *j*) or *S*_12_(*i*, *j*) as described above. The crystal growth rate is estimated by the crystal growth distance normal to the initial interface and the corresponding time elapsed.

### Diffusion coefficient

The diffusion coefficient *D*(*T*) is calculated from the mean-squared displacements

15 $$\left\langle \Delta {r}^{2}(t)\right\rangle=\frac{1}{N}\langle \sum {\left[{{{{{{{\bf{r}}}}}}}}(t)-{{{{{{{\bf{r}}}}}}}}(0)\right]}^{2}\rangle$$

as

16 $$D(T)=\mathop{\lim }\limits_{t\to \infty }\frac{\left\langle \Delta {r}^{2}(t)\right\rangle }{6t}.$$

To measure *D*(*T*), we equilibrate the liquids at each temperature and then output atomic trajectories to calculate the mean-squared displacements (see examples in Supplementary Fig. 13). In more detail, we first equilibrate the liquid at a temperature much higher than *T*_m_ and then bring the configuration to the target temperature for further equilibration. The ensemble will then be switched from *NPT* to *NVT* (constant number *N*, constant volume *V*, constant temperature *T*) for production runs. We use *N* = 11664 atoms for these calculations. The configurations from these simulations are also used to analyse the structural orderings in the equilibrated supercooled liquids.

## Supplementary information

Supplementary Information

## Data Availability

The data that support the findings of this study are available from the corresponding author upon reasonable request.

## Code availability

The simulation codes used in this study are available from the corresponding authors on reasonable request.

## References

[1] Kelton, K. F. & Greer, A. L. *Nucleation in condensed matter: applications in materials and biology* (Pergamon, 2010).

[2] Auer S, Frenkel D (2001). Prediction of absolute crystal-nucleation rate in hard-sphere colloids. Nature.

[3] Gasser U, Weeks ER, Schofield A, Pusey PN, Weitz DA (2001). Real-space imaging of nucleation and growth in colloidal crystallization. Science.

[4] Kawasaki T, Tanaka H (2010). Formation of a crystal nucleus from liquid. Proc. Natl. Acad. Sci. U.S.A..

[5] Russo J, Tanaka H (2016). Nonclassical pathways of crystallization in colloidal systems. MRS Bull..

[6] Savage JR, Dinsmore AD (2009). Experimental evidence for two-step nucleation in colloidal crystallization. Phys. Rev. Lett..

[7] Tan P, Xu N, Xu L (2014). Visualizing kinetic pathways of homogeneous nucleation in colloidal crystallization. Nat. Phys..

[8] Arai S, Tanaka H (2017). Surface-assisted single-crystal formation of charged colloids. Nat. Phys..

[9] Sosso GC (2016). Crystal nucleation in liquids: open questions and future challenges in molecular dynamics simulations. Chem. Rev..

[10] Li M, Chen Y, Tanaka H, Tan P (2020). Revealing roles of competing local structural orderings in crystallization of polymorphic systems. Sci. Adv..

[11] Yoreo JJD (2015). Crystallization by particle attachment in synthetic, biogenic, and geologic environments. Science.

[12] Ostwald W (1897). The formation and changes of solids. Z. Phys. Chem..

[13] Stranski IN, Totomanow D (1933). Rate of formation of (crystal) nuclei and the Ostwald step rule. Z. Phys. Chem..

[14] Alexander S, McTague J (1978). Should all crystals be bcc? Landau theory of solidification and crystal nucleation. Phys. Rev. Lett..

[15] ten Wolde PR, Ruiz-Montero MJ, Frenkel D (1995). Numerical evidence for bcc ordering at the surface of a critical fcc nucleus. Phys. Rev. Lett..

[16] Tanaka H (2012). Bond orientational order in liquids: Towards a unified description of water-like anomalies, liquid-liquid transition, glass transition, and crystallization. Eur. Phys. J. E.

[17] Becker S, Devijver E, Molinier R, Jakse N (2022). Unsupervised topological learning for identification of atomic structures. Phys. Rev. E.

[18] Russo J, Tanaka H (2012). The microscopic pathway to crystallization in supercooled liquids. Sci. Rep..

[19] Russo J, Tanaka H (2016). Crystal nucleation as the ordering of multiple order parameters. J. Chem. Phys..

[20] Greer AL (2015). New horizons for glass formation and stability. Nat. Mater..

[21] Sun G, Xu J, Harrowell P (2018). The mechanism of the ultrafast crystal growth of pure metals from their melts. Nat. Mater..

[22] Hawken A, Sun G, Harrowell P (2019). Role of interfacial inherent structures in the fast crystal growth from molten salts and metals. Phys. Rev. Mater..

[23] Sun G, Hawken A, Harrowell P (2020). The displacement field associated with the freezing of a melt and its role in determining crystal growth kinetics. Proc. Natl. Acad. Sci. USA.

[24] Gao Q (2021). Fast crystal growth at ultra-low temperatures. Nat. Mater..

[25] Tanaka H, Tong H, Shi R, Russo J (2019). Revealing key structural features hidden in liquids and glasses. Nat. Rev. Phys..

[26] Wilson HW (1900). XX. On the velocity of solidification and viscosity of super-cooled liquids. Philos. Mag..

[27] Frenkel J (1946). Kinetic theory of liquids.

[28] Tanaka H (2003). Roles of local icosahedral chemical ordering in glass and quasicrystal formation in metallic glass formers. J. Phys.: Condens. Matter.

[29] Jakse N, Pasturel A (2003). Local order of liquid and supercooled zirconium by ab initio molecular dynamics. Phys. Rev. Lett..

[30] Kelton KF (2003). First x-ray scattering studies on electrostatically levitated metallic liquids: demonstrated influence of local icosahedral order on the nucleation barrier. Phys. Rev. Lett..

[31] Cheng YQ, Ma E (2011). Atomic-level structure and structure-property relationship in metallic glasses. Prog. Mater. Sci..

[32] Hirata A (2011). Direct observation of local atomic order in a metallic glass. Nat. Mater..

[33] Yuan Y (2022). Three-dimensional atomic packing in amorphous solids with liquid-like structure. Nat. Mater..

[34] Frank FC (1952). Supercooling of liquids. Proc. R. Soc. A.

[35] Wu ZW (2016). Critical scaling of icosahedral medium-range order in CuZr metallic glass-forming liquids. Sci. Rep..

[36] Desgranges C, Delhommelle J (2018). Unusual crystallization behavior close to the glass transition. Phys. Rev. Lett..

[37] Hu Y-C (2020). Glass formation in binary alloys with different atomic symmetries. Phys. Rev. Mater..

[38] Hu Y-C, Tanaka H (2020). Physical origin of glass formation from multicomponent systems. Sci. Adv..

[39] Wu DT, Gránásy L, Spaepen F (2004). Nucleation and the solid-liquid interfacial free energy. MRS Bull..

[40] Gránásy L, Iglói F (1997). Comparison of experiments and modern theories of crystal nucleation. J. Chem. Phys..

[41] Turnbull D (1950). Formation of Crystal Nuclei in Liquid Metals. J. Appl. Phys..

[42] Aga RS, Morris JR, Hoyt JJ, Mendelev M (2006). Quantitative parameter-free prediction of simulated crystal-nucleation times. Phys. Rev. Lett..

[43] Keys AS, Glotzer SC (2007). How do quasicrystals grow?. Phys. Rev. Lett..

[44] Shen YT, Kim TH, Gangopadhyay AK, Kelton KF (2009). Icosahedral order, frustration, and the glass transition: evidence from time-dependent nucleation and supercooled liquid structure studies. Phys. Rev. Lett..

[45] Inoue A (1998). Amorphous, nanoquasicrystalline and nanocrystalline alloys in Al-based systems. Prog. Mater. Sci..

[46] Russo J, Romano F, Tanaka H (2018). Glass forming ability in systems with competing orderings. Phys. Rev. X.

[47] Desgranges C, Delhommelle J (2014). Unraveling the coupling between demixing and crystallization in mixtures. J. Am. Chem. Soc..

[48] Puosi F, Jakse N, Pasturel A (2018). Dynamical, structural and chemical heterogeneities in a binary metallic glass-forming liquid. J. Phys.: Condens. Matter.

[49] Desgranges C, Delhommelle J (2019). Can ordered precursors promote the nucleation of solid solutions?. Phys. Rev. Lett,.

[50] Ingebrigtsen TS, Dyre JC, Schrøder TB, Royall CP (2019). Crystallization instability in glass-forming mixtures. Phys. Rev. X.

[51] Zhong L, Wang J, Sheng H, Zhang Z, Mao SX (2014). Formation of monatomic metallic glasses through ultrafast liquid quenching. Nature.

[52] Mishin Y, Mehl MJ, Papaconstantopoulos DA (2002). Embedded-atom potential for B2-NiAl. Phys. Rev. B.

[53] Plimpton S (1995). Fast parallel algorithms for short-range molecular dynamics. J. Comput. Phys..

[54] Levchenko EV, Evteev AV, Belova IV, Murch GE (2011). Molecular dynamics determination of the time-temperature-transformation diagram for crystallization of an undercooled liquid Ni50Al50 alloy. Acta Mater..

[55] Tribello GA, Bonomi M, Branduardi D, Camilloni C, Bussi G (2014). PLUMED 2: New feathers for an old bird. Comput. Phys. Commun..

[56] Steinhardt PJ, Nelson DR, Ronchetti M (1983). Bond-orientational order in liquids and glasses. Phys. Rev. B.

[57] Rycroft CH (2009). VORO++: A three-dimensional Voronoi cell library in C++. Chaos.

[58] Mickel W, Kapfer SC, Schröder-Turk GE, Mecke K (2013). Shortcomings of the bond orientational order parameters for the analysis of disordered particulate matter. J. Chem. Phys..

[59] Leocmach M, Tanaka H (2012). Roles of icosahedral and crystal-like order in the hard spheres glass transition. Nat. Commun..

[60] Mendelev MI (2009). Development of suitable interatomic potentials for simulation of liquid and amorphous Cu-Zr alloys. Philos. Mag..

[61] Foiles SM, Baskes MI, Daw MS (1986). Embedded-atom-method functions for the fcc metals Cu, Ag, Au, Ni, Pd, Pt, and their alloys. Phys. Rev. B.

[62] Molinero V, Sastry S, Angell CA (2006). Tuning of tetrahedrality in a silicon potential yields a series of monatomic (metal-like) glass formers of very high fragility. Phys. Rev. Lett..

[63] Molinero V, Moore EB (2009). Water modeled as an intermediate element between carbon and silicon. J. Phys. Chem. B.

