# Supplementary material for: Revealing the role of liquid preordering in crystallisation of supercooled liquids
Source: Nat Commun. 2022 Aug 4;13:4519. doi: 10.1038/s41467-022-32241-z (PMC9352720; doi:10.1038/s41467-022-32241-z)
Supplement: Supplementary file 1 — Supplementary Information [file 41467_2022_32241_MOESM1_ESM.pdf]

Supplementary Information for "Revealing the role of liquid preordering in crystallisation of supercooled liquids"

Yuan-Chao Hu[1] and Hajime Tanaka[1,2,*]

[1]*Department of Fundamental Engineering, Institute of Industrial Science, University of Tokyo, 4-6-1 Komaba, Meguro-ku, Tokyo 153-8505, Japan*

[2]*Research Center for Advanced Science and Technology, University of Tokyo, 4-6-1 Komaba, Meguro-ku, Tokyo 153-8904, Japan*

[*] E-mail: tanaka@iis.u-tokyo.ac.jp

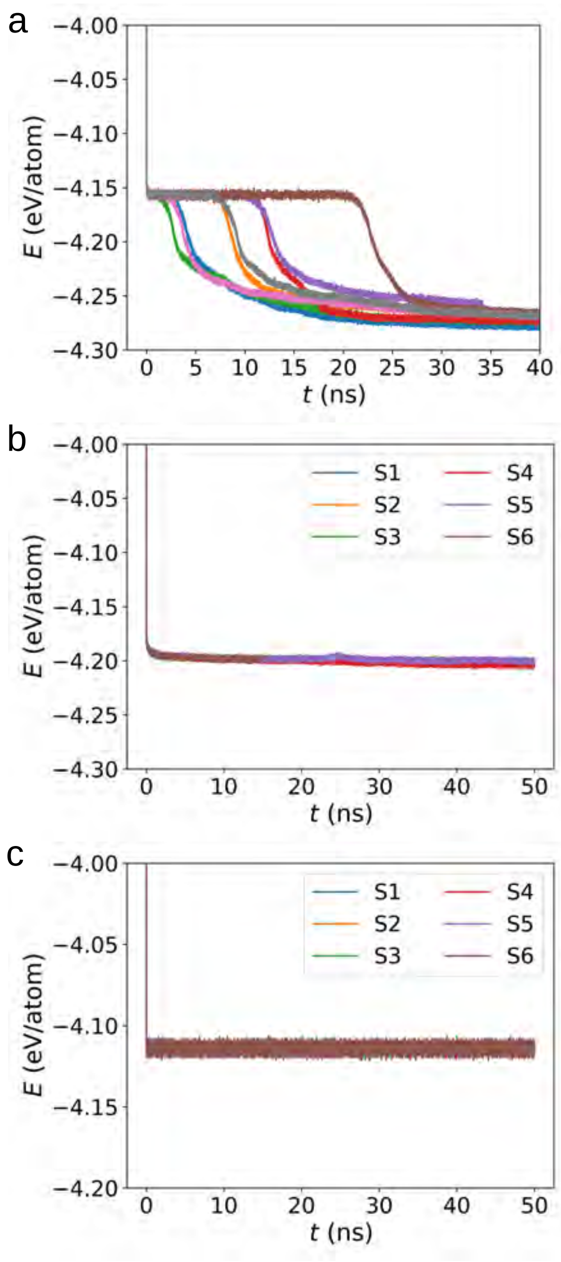

Supplementary Fig. 1. **Potential energy during isothermal annealing for NiAl**. **a,** At T_{nose}, which is around $0.6T_{\mathrm{m}}$. The data shown are for 8 independent simulations. The discontinuous jump in the potential energy indicates crystallisation. **b-c,** At $0.5T_{\mathrm{m}}$ and $0.7T_{\mathrm{m}}$, respectively. 6 independent simulations are carried out in **b** and **c**, but no one shows crystallisation. This indicates T_{nose} is indeed around $0.6T_{\mathrm{m}}$.

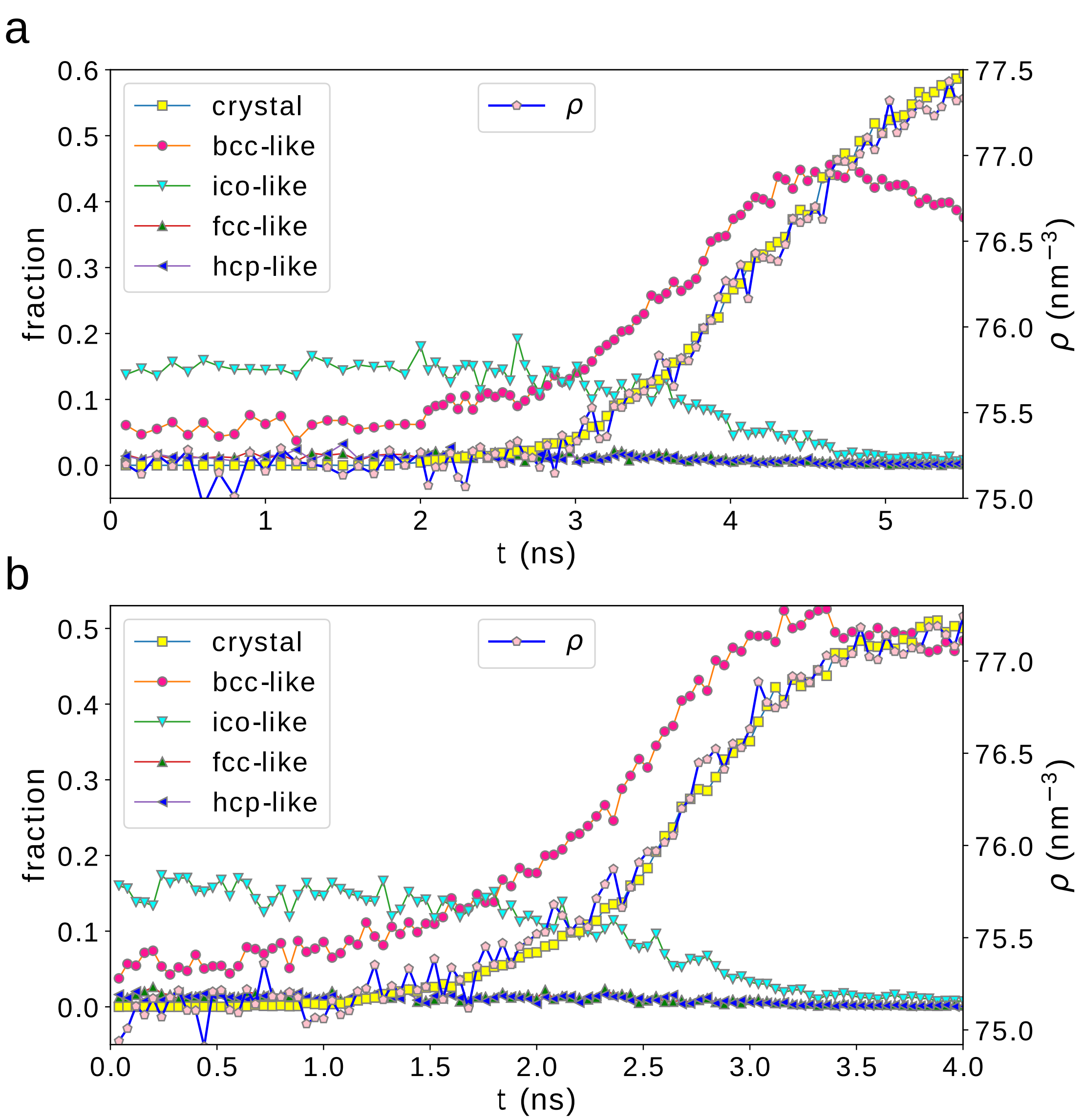

Supplementary Fig. 2. **The time evolutions of the fractions of crystallized atoms and different types of structural orders in the two crystallization processes.** The number density change is included for comparison with the crystallised atoms (crystal), different crystal-like orders (bcc-like, fcc-like, and hcp-like), and icosahedral-like orders. The two plots are from two independent simulations at the same condition as Fig. 1a, showing the robustness of the basic features shown in Fig. 1a in the main text.

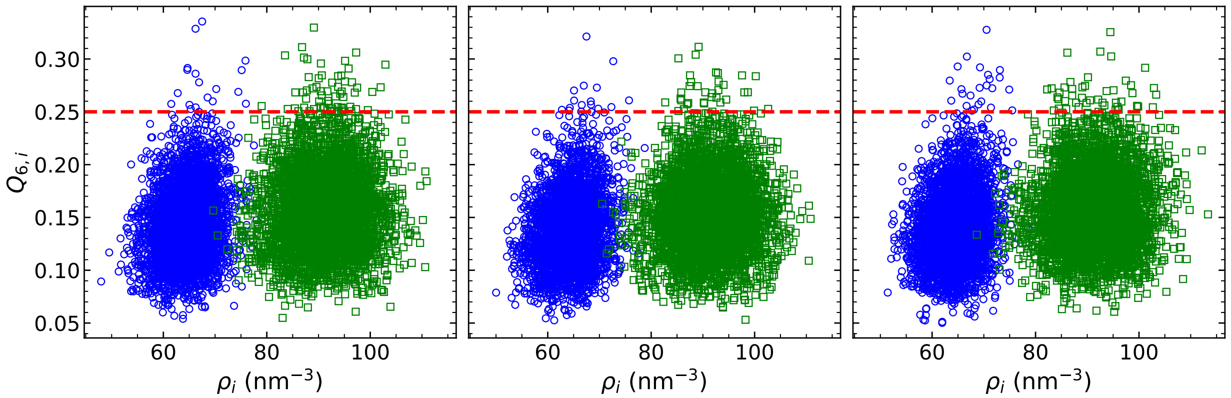

Supplementary Fig. 3. **Correlation maps between the particle level density ρ_i and $Q_{6,i}$ at three different timescales in the supercooled metallic liquid before crystallisation occurs.** The two groups from ρ_i are for different species, Ni (green squares) or Al (blue circles). The centres of bcc-like preorder typically with $Q_{6,i} > 0.25$ (marked as red dashed line) have a wide local density distribution for both species. This demonstrates little coupling between the two fields in the supercooled liquid.

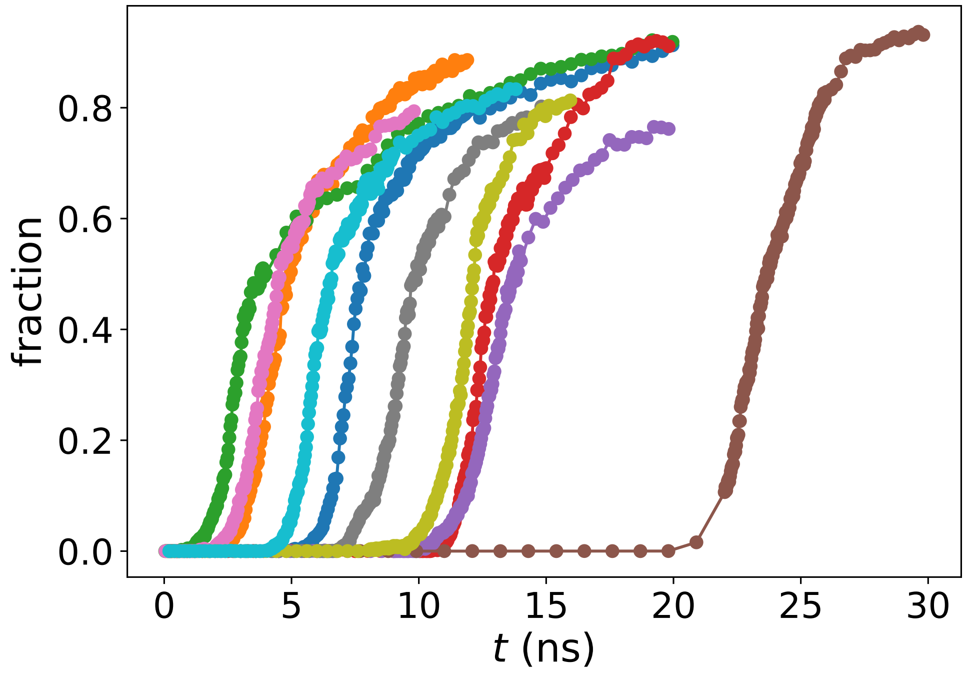

Supplementary Fig. 4. **The fraction of crystallised atoms during the crystallization process for 10 independent simulations for NiAl at** T_{nose}. We identify the time when the crystal nucleus size exceeds the critical one, i.e., the crystal nucleation time, as the time when 1% atoms crystallise.

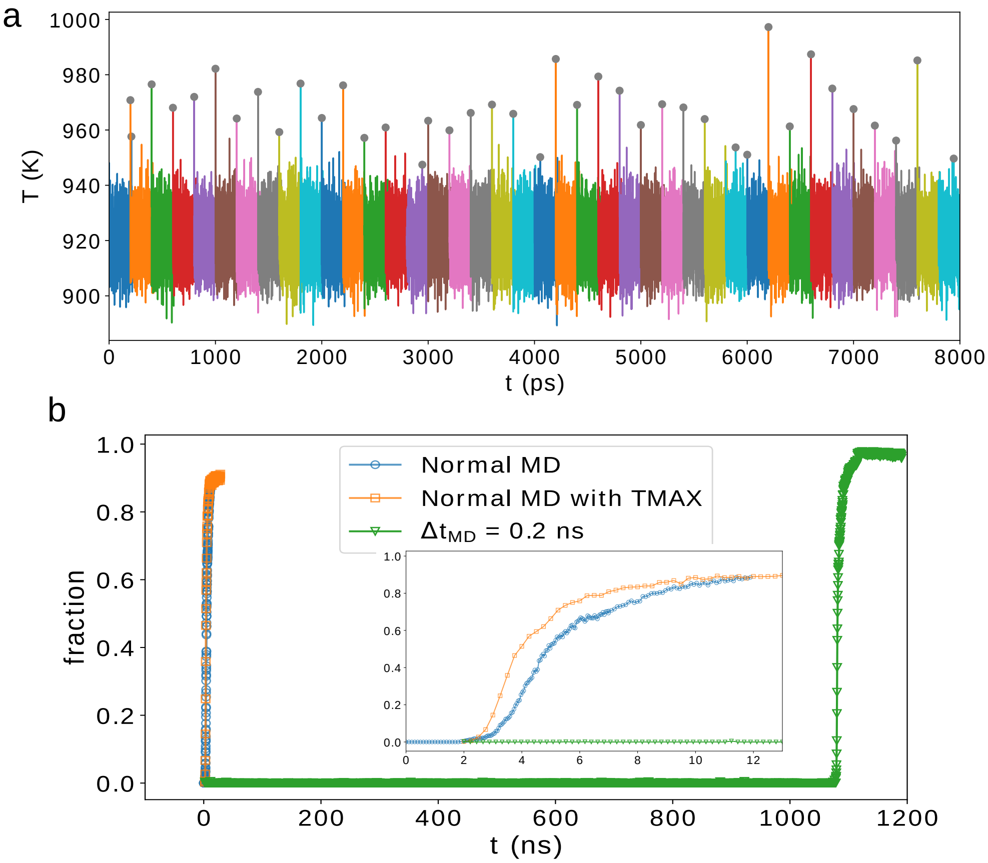

Supplementary Fig. 5. Effect of the temperature change induced by the bias potential on crystal nucleation for NiAl. a, The temperature change in simulations (at $T_{\mathrm{nose}} = 921$ K) caused by a bias potential implemented. The temperature change duration is almost instantaneous. The peak temperature during the biasing in each cycle is marked as the grey circle. We refer to the average peak temperature as TMAX. TMAX may be regarded as the upper bound of the effective temperature of our hybrid simulation. **b,** The comparison of the crystal nucleation time between the normal MD simulation at TMAX ($> T_{\mathrm{nose}}$) and the ones at T_{nose} with and without OKS. The inset shows the results at a shorter timescale. It is evident that the nucleation times of the normal MD simulations at T_{nose} and at TMAX are about the same, but they are remarkably shorter than the one with OKS. We can see that killing crystal-like preorder delays crystallisation significantly, but the slight increase in the temperature accelerates crystallisation only slightly. This result proves that killing crystal-like preorder in the supercooled liquid state is critical for impeding crystal nucleation, and the temperature change induced by biasing is not responsible for it.

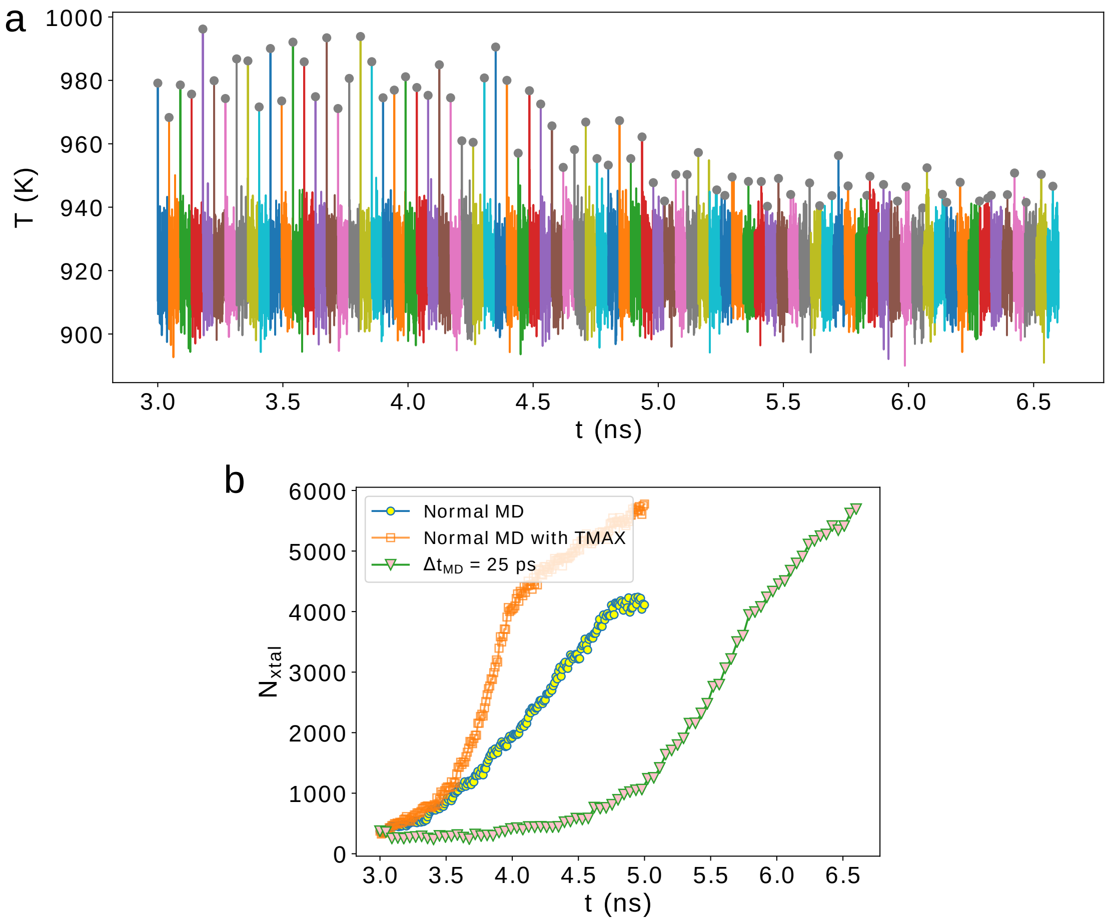

Supplementary Fig. 6. Effect of the temperature change induced by the bias potential on crystal growth for NiAl. a, The temperature change in simulations (at $T_{\rm nose} = 921$ K) caused by a bias potential implemented. As in the above case of crystal nucleation, the temperature change duration is almost instantaneous. The peak temperature in each cycle of killing order is marked as the grey circle. The peak temperature decreases with the decrease of the number of preorders as crystallisation proceeds. We again refer to the average peak temperature as TMAX. TMAX may be regarded as the upper bound of the effective temperature of our hybrid simulation. **b,** The comparison of the increasing number of crystallised atoms between the normal MD simulation at TMAX and the ones at $T_{\rm nose}$ with and without OKS. It is obvious that the slight increase in the temperature can accelerate crystal growth, but killing crystal-like preorder can effectively impede crystal growth. Note that the slope of OKS at intermediate $N_{\rm xtal}$ is smaller than that of the normal MD at TMAX. This result proves that killing crystal-like preorder in the supercooled liquid state is critical for impeding crystal growth, and the temperature change induced by biasing is not responsible for it.

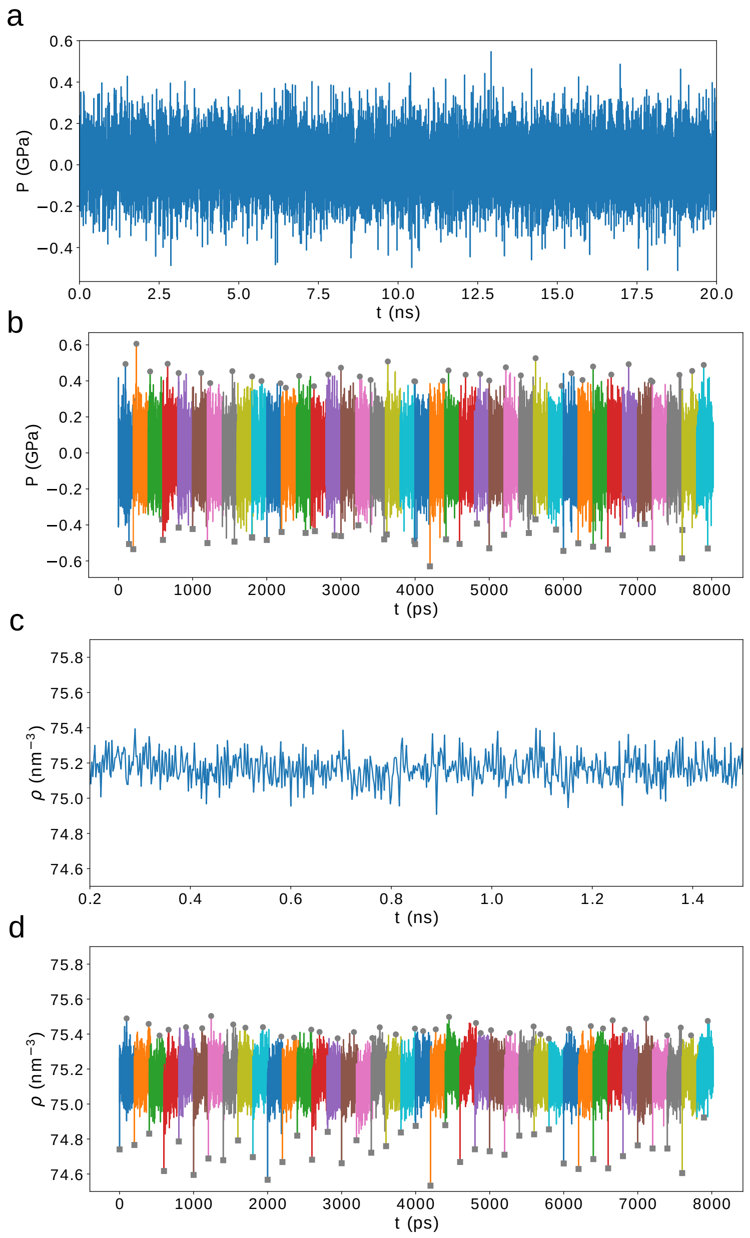

Supplementary Fig. 7. Effects of the bias potential on the fluctuations of the pressure and number density for NiAl at $T_{\rm nose}$. a,b, Temporal fluctuations of the pressure in normal MD simulations covering the full time range from a liquid state to a crystallised state (**a**) and those in the hybrid simulations (**b**) as in Supplementary Fig. 5. The average pressure is almost not affected and remains almost 0. **c,d,** Temporal fluctuations of the number density in normal MD simulations in the time range covering only the liquid state (**c**) and those in the hybrid simulations (**d**) as in Supplementary Fig. 5. As for the number density, the average is the same between (**c**) and (**d**). However, there are larger fluctuations in the number density with the biasing potential, consistent with the larger temperature fluctuation (see Supplementary Fig. 5) since the pressure is almost not affected by the bias potential.

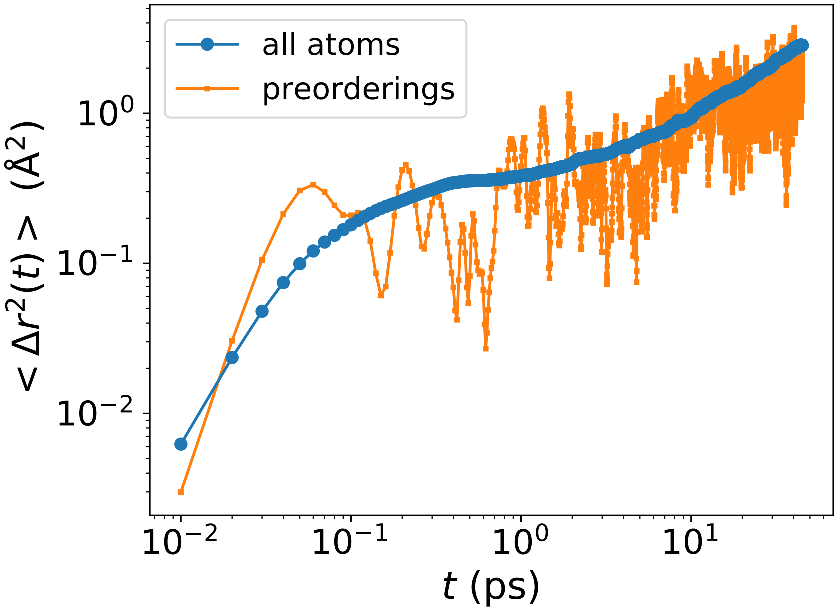

Supplementary Fig. 8. **Effect of the bias potential on the atomic motion.** The bias potential driving order killing adjusts the centre particle's position only within its cage. Thus, it affects only the ballistic regime of atomic motion in a preorder. We show only one period of the biasing for comparison between with and without biasing.

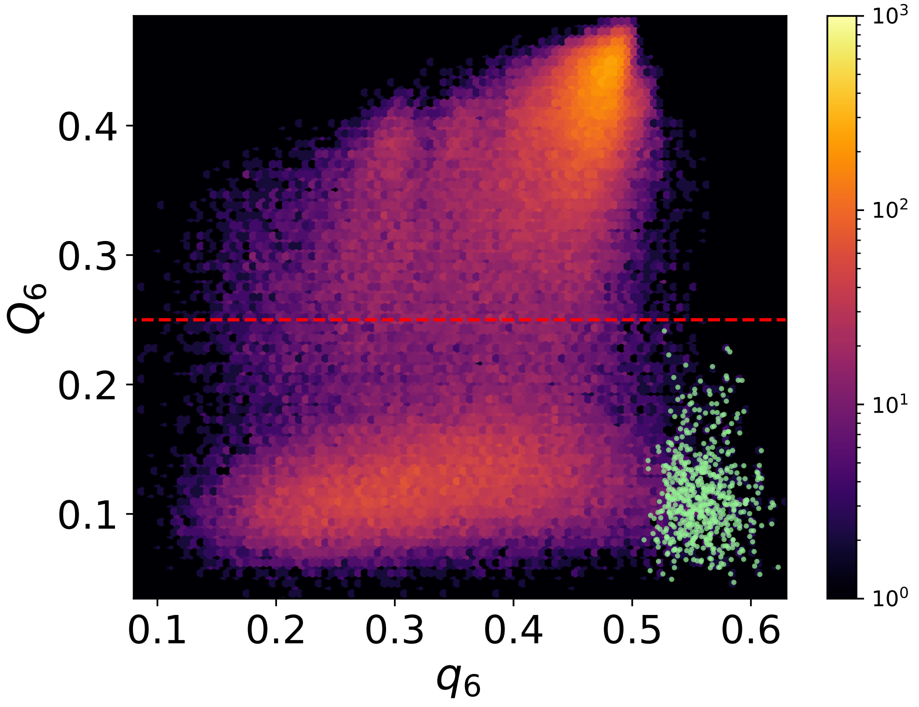

Supplementary Fig. 9. Q_6-q_6 **map in the supercooled liquid of NiAl at** $T_{\rm nose}$. The dashed line shows $Q_6 = 0.25$, which is used to define the preorderings to kill. The green points represent atoms showing local icosahedral order identified by w_6.

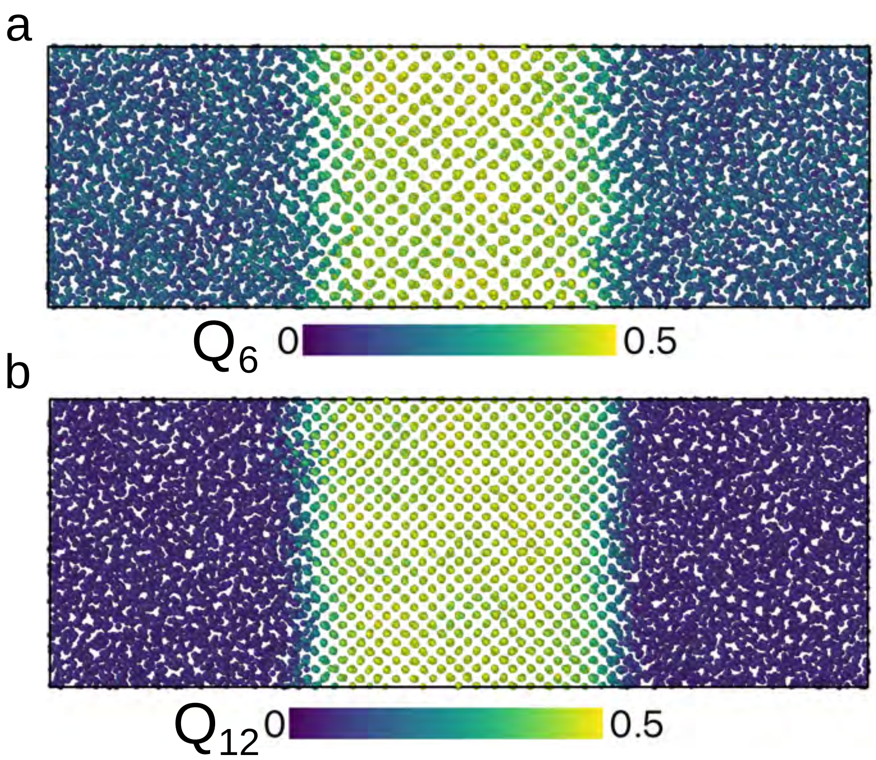

Supplementary Fig. 10. **Examples of the liquid-crystal interface used to evaluate the crystal growth rate. a,** Ta at $0.6T_{\mathrm{m}}$ with atoms coloured with Q_6. **b,** Si at $0.6T_{\mathrm{m}}$ with atoms coloured with Q_{12}. In both cases, the crystal and liquid phases are well differentiated by the chosen order parameters. The order parameter profile around the interface demonstrates the wetting of particles with high bond orientational order to the crystal growth front. We can see that the liquid-crystal interfaces are much sharper for Si than Ta. The smaller contrast between the liquid and solid in Ta indicates its stronger tendency of forming crystal-like preordering, helping crystallisation.

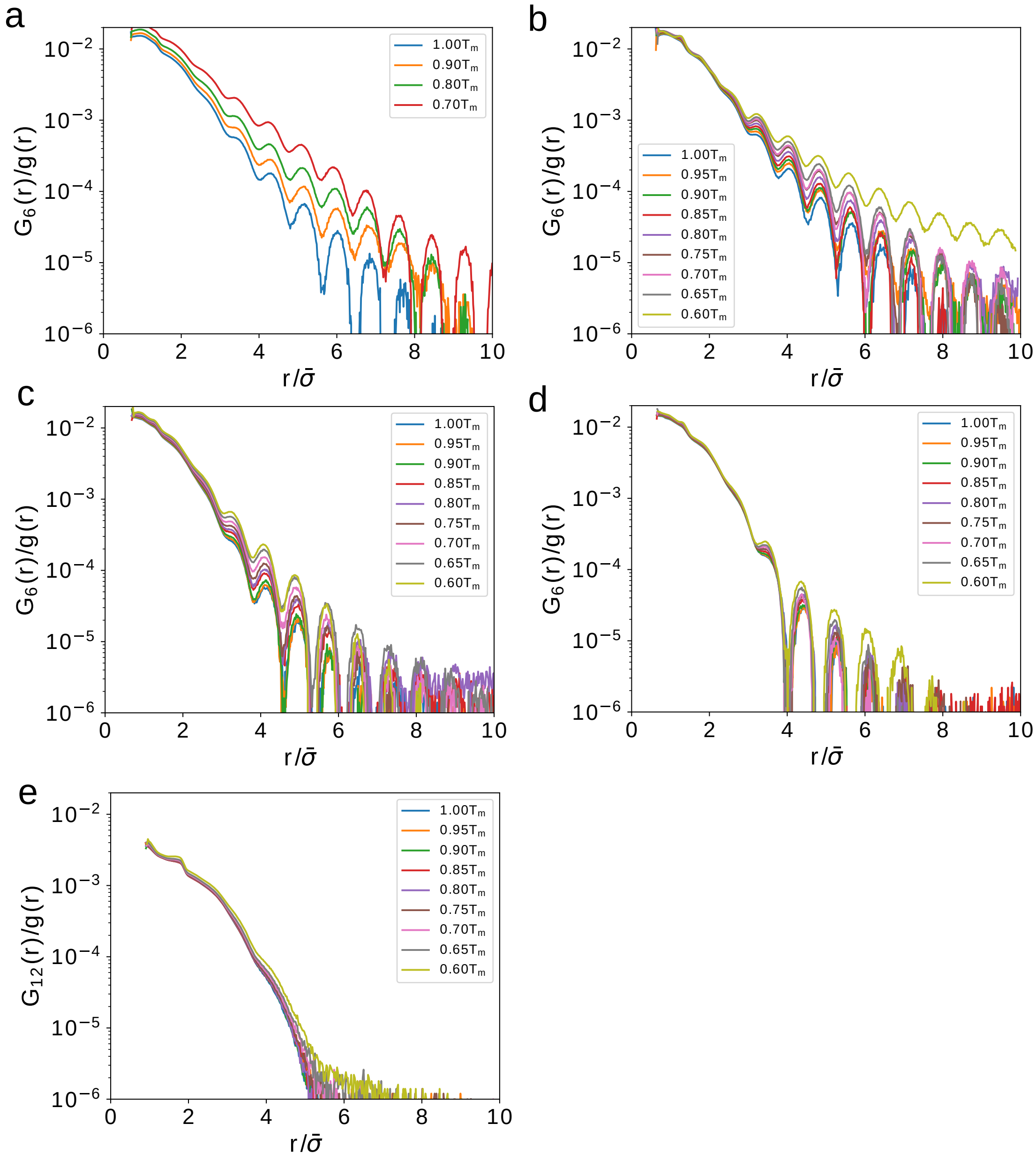

Supplementary Fig. 11. **Spatial correlations of the bond orientational order parameters.** The system in **a-e** is Zr, Ta, NiAl, CuZr, and Si, respectively. $\bar{\sigma}$ is the (average) diameter of each system. The order parameter is Q_6 for Zr, Ta, NiAl, and CuZr , whereas Q_{12} for Si. The spatial decay of the correlation function is shown for each system from T_{m} to $0.6T_{\mathrm{m}}$ except Zr for which to $0.7T_{\mathrm{m}}$. The vertical and horizontal axes ranges are set as the same for all the systems for comparison. From **a** to **e**, the spatial correlation of the order parameter becomes weaker. Especially in Si, there is almost no correlation. The stronger spatial correlation of the crystal-like order parameter in a supercooled liquid should assist crystal growth from the topological viewpoint. On the other hand, chemical frustration should also play a role in crystal growth for binary systems.

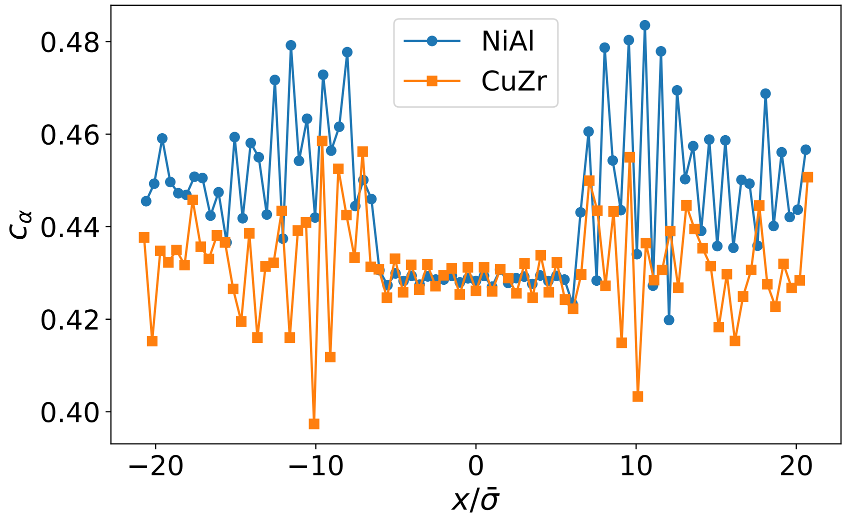

Supplementary Fig. 12. **Density profiles along the liquid-crystal interfaces at $0.7T_\mathrm{m}$.** The distribution of the local composition c_α is shown near the interface in NiAl and CuZr. For perfect bcc-like crystals, $c_\alpha = 6/14 \approxeq 0.43$. The small fluctuation in the crystalline phase is because of the thermal noise. Both systems suffer from some compositional frustration at the interface front but behaving differently depending on the interatomic interactions. Combing the analyses of structural ordering and local composition, we can easily find that all the factors, including the amount, the spatial correlation, and the composition of the preorders, are crucial in determining the interfacial properties and thus the crystal growth rate.

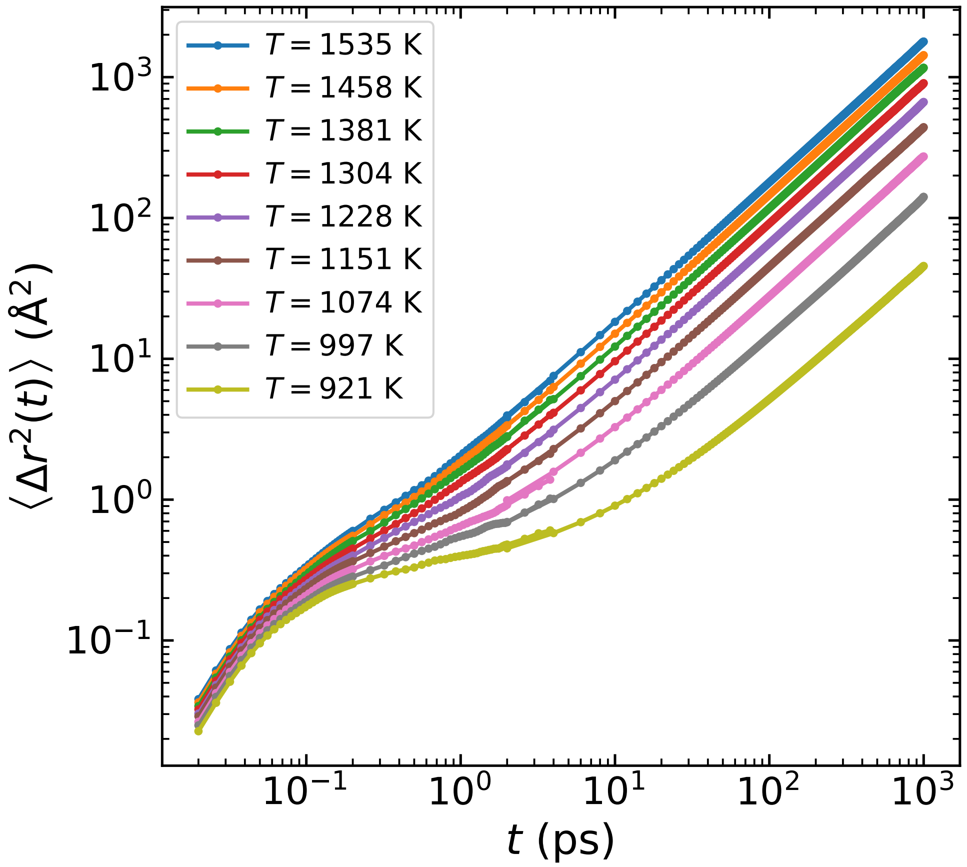

Supplementary Fig. 13. **Mean-squared displacements at different temperatures for NiAl.** The long-time diffusive regime is used to estimate the diffusion coefficient.
